# No free lunch in ball catching: A comparison of Cartesian and angular representations for control

**DOI:** 10.1371/journal.pone.0197803

**Published:** 2018-06-14

**Authors:** Sebastian Höfer, Jörg Raisch, Marc Toussaint, Oliver Brock

**Affiliations:** 1 Robotics and Biology Laboratory (RBO), Technische Universität Berlin, Berlin, Germany; 2 Amazon Research, Berlin, Germany; 3 Control Systems Group, Technische Universität Berlin, Berlin, Germany; 4 Machine Learning and Robotics Lab (MLR), Universität Stuttgart, Stuttgart, Germany; Berner Fachhochschule, SWITZERLAND

## Abstract

How to run most effectively to catch a projectile, such as a baseball, that is flying in the air for a long period of time? The question about the best solution to the ball catching problem has been subject to intense scientific debate for almost 50 years. It turns out that this scientific debate is not focused on the ball catching problem alone, but revolves around the research question what constitutes the ingredients of intelligent decision making. Over time, two opposing views have emerged: the *generalist* view regarding intelligence as the ability to solve any task without knowing goal and environment in advance, based on optimal decision making using predictive models; and the *specialist* view which argues that intelligent decision making does not have to be based on predictive models and not even optimal, advocating simple and efficient rules of thumb (*heuristics*) as superior to enable accurate decisions. We study two types of approaches to the ball catching problem, one for each view, and investigate their properties using both a theoretical analysis and a broad set of simulation experiments. Our study shows that neither of the two types of approaches can be regarded as superior in solving all relevant variants of the ball catching problem: each approach is optimal under a different realistic environmental condition. Therefore, predictive models neither guarantee nor prevent success a priori, and we further show that the key difference between the generalist and the specialist approach to ball catching is the type of input representation used to control the agent. From this finding, we conclude that the right solution to a decision making or control problem is orthogonal to the generalist and specialist approach, and thus requires a reconciliation of the two views in favor of a representation-centric view.

## 1 Introduction

There is a long standing debate about what constitutes the ingredients of intelligent decision making. Over time, two opposing views have emerged, both supported by a broad body of scientific work. On the one hand, there is what we call the *generalist* view: it regards intelligence as the ability to solve any task without knowing goal and environment in advance [1, 2]. A key feature of many generalist agents is an internal predictive model of the environment. The predictive model can either be learned from experience, or, as done in many practical applications, is defined manually and provided to the agent. In both cases, optimal decisions based on the model are inferred in order to progress towards the goal.

On the other hand, there is the *specialist* view. It argues that intelligent decision making does not have to be based on predictive models (“as-if models”, [3]) and not even optimal [4]. Instead, simple and efficient rules of thumb enable accurate decisions: *heuristics*. Proponents of this view argue that heuristics are better-suited for complex decision making because the real world is too complex and exhibits too much uncertainty in order to be modeled precisely.

In this paper, we study one of the most prominent examples in the debate between specialists and generalists: the outfielder ball catching problem [5]. This problem deals with the question of how to run most effectively to intercept a moving target, such as a baseball that is flying in the air for a long period of time. Despite the significance and ubiquity of this capability in humans and animals, for example for intercepting prey, specialists and generalists still debate about the biologically most plausible and the most effective strategy to solve this problem [6]. Therefore, understanding the computational principles behind ball interception may yield important insights about which view, generalist or specialist, is better-suited to account for many problems in intelligent decision making. *Generalists* argue that the problem is solved most effectively by estimating the ball’s position and velocity, predicting the ball’s trajectory and running towards the landing point. *Specialists* claim that the agent intercepts the ball most effectively if it relies on a heuristic based on the angle between the ground plane and the ball as seen by the agent [6–8]—ignoring all causal variables necessary to compute the trajectory of the ball, such as the ball’s position and velocity.

The main finding of this paper is that **(i) neither the generalist nor specialist approach is superior in solving all relevant variants of the ball catching problem: each approach is optimal under a different realistic environmental condition**. From this finding, we conclude that research on intelligent decision making should not dichotomize the generalist and specialist approaches, but study their individual strengths and investigate how to combine them. Our paper identifies their strengths by providing a thorough mathematical treatment as well as an extensive empirical evaluation in the context of ball catching, and thus makes an important step towards unifying the two approaches for general intelligent decision making problems.

Our main finding that neither of the two approaches is superior, has a variety of important implications on the study of the ball catching problem as well as on the study of decision making problems in general:

**(ii) Predictive models neither guarantee nor prevent success a priori**.

Our study shows that the generalist, model-based approach is superior in coping with high-frequency perturbations of the ball trajectory, such as perceptual or motor noise. However, it fails when the model’s assumptions are systematically violated, for example, when air resistance affects the ball trajectory but the model neglects it. In contrast, the specialist, model-free approach is able cope better with such model violations, but cannot cope with high-frequency noise.

Our finding is well-supported by previous work on reinforcement learning [9] where both model-free and model-based approaches are common, and both are known to each have their strengths and weaknesses. Therefore, we should refrain from favoring or rejecting model-based approaches a priori for solving decision making problems.

**(iii) The specialist’s ball catching heuristic is an optimal solution under certain environmental conditions**.

Despite advocating task-specificity, the specialist view should not be misinterpreted as regarding heuristics to be opposed to optimization. On the contrary, proponents of the specialist view have shown that heuristics are optimal solutions in single decision making problems (classification/regression) with low amounts of training data and high-uncertainty [10, 11]. Our study shows that the same result holds true for ball catching, and thus for *sequential* decision making problems: we show that the generalist approach to ball catching can be viewed as optimal in the presence of high-frequency Gaussian noise, and the specialist approach as optimal in the absence of such noise. Therefore, optimality is not a criterion that distinguishes generalist and specialist approaches for decision making.

**(iv) The key difference between the generalist and the specialist approach to ball catching is the type of input representation used to control the agent**.

Our results raise the question why the two approaches perform different under varying environmental conditions, and whether this difference is due to the use of a predictive model. To answer this question, we investigate whether the specialist, heuristic approach can be turned into a model-based approach. We prove that this is not the case: the input representation used by the heuristic is not amenable to predictive modeling as it does not fulfill the Markov assumption required by most model-based approaches. Therefore, we conclude that the input representation used to control the agent is the key factor for the applicability of a model-free or model-based approach, and thus performance under different environmental conditions.

Given these findings, we argue that the generalist and specialist approach form two extreme ends of a spectrum. We conjecture that combining the two approaches by moving along that spectrum will help us to find better representations, and thus better solutions for both the ball catching problem under all realistic environmental conditions as well as other decision making problems.

### 1.1 Outline

The outline of this paper is the following. We begin by formalizing the ball catching problem in Section 2. We introduce the generalist and specialist approaches to this problem, highlighting that the generalist strategies rely on a *Cartesian representation* whereas the heuristic strategies are based on an *angular representation* of the ball position with respect to the agent. For both approaches we discuss prior work (Section 3), derive suitable control implementations for controllers suggested in previous literature as well as novel ones, study their mathematical properties (Section 4.1 and Section 4.2) and evaluate them in a extensive set of empirical simulation experiments (Section 4.4). We then present the main results of our work, namely that certain environmental conditions affect the specialist more severely than the generalist strategies—and vice versa. All of our findings are both, derived theoretically and confirmed by simulation experiments. To draw further conclusions from our main result, we investigate the role of optimality and predictive models for distinguishing between generalist and specialist approaches for ball catching. First, we show that the angular representation is not amenable to similar types of predictive modeling as the Cartesian representation (Section 4.3). Second, we show that the specialist, heuristic solution to ball catching results from applying model-free reinforcement learning under the heuristic’s preferred environmental conditions (Section 5). Section 6 discusses the implications of our findings for future research on ball catching in particular and decision making in general in. Section 7 concludes the paper.

### 1.2 Technical contributions

In the following, we detail our work’s technical contributions:

#### 1.2.1 Theoretical results

1. We formally show that different implementations of *Chapman’s strategy* [5], one of the main heuristic strategies for the simplified two-dimensional ball catching problem, all form *a unified mathematical framework*. They only differ with respect to the type of derivative of the *angular representation* used as a control signal (Section 4.2).
2. We prove that Chapman’s strategy generates an agent reference trajectory that, if attained by a controller, guarantees interception for both *ideal flight trajectories* and *systematically perturbed trajectories*. This includes perturbations that resemble air resistance (Section 4.2).
3. We further show that Chapman’s strategy *does not require computation of the angle’s acceleration* (the second derivative) but only of its velocity. This insight allows us *to develop two robust variants of angle-based controllers: COV-IO and COV-OAC* (Section 4.3).
4. We show that the angular representation employed by Chapman’s strategy results in a *stationary control problem* and can be implemented with bang-bang control (Section 4.3).
5. Our analysis shows that this simplified control scheme comes at the cost of intractable dynamics: *none of the angular representations used for control is Markov* (Section 4.3.8), and thus no closed-form description of the dynamics based on the angular representation exists.
6. In contrast, we show that a *Cartesian representation* of agent and ball results in *simpler dynamics* (linear for ideal parabolic flight, locally linear for air resistance). This comes at the expense of *non-stationary PD-control* required to catch accurately (Section 4.1).

#### 1.2.2 Empirical results

1. An evaluation of a wide variety of systematic and random perturbations shows that *Chapman’s strategy generalizes over systematic perturbations of the ball trajectory, such as air resistance, whereas strategies based on the Cartesian representation generalize better over perturbations induced by Gaussian noise*, both in two and three dimensions (Section 4.4).
2. When formulating ball catching in two dimensions as a model-free reinforcement learning problem, searching for a policy that directly maps from camera sensor input to control output, a generic *black-box optimization with regularization learns a policy equivalent to Chapman’s strategy* (Sec. 5).

## 2 Formal statement of the ball catching problem

In this section, we formalize the ball catching problem and sketch the solution approaches suggested by the generalist and specialist camps.

The task of ball catching, and more generally *projectile interception*, is arguably one of the most studied problems in psychology and control (see Section 3). Interception involves a variety of different sub-skills, such as visually perceiving the target, fixating it, running towards it, as well as moving arm and hand in a suitable manner. In this work, we focus on the question of how to run towards a target in order to intercept it; the problem is therefore reduced to finding the motion of an agent on the two-dimensional (ground) plane. Moreover, we confine ourselves to a passive projectile, for example a baseball (rather than a rocket or bird). In an idealized setting, the trajectory of a ball can be fully predicted from its initial position and velocity; however, empirical studies have shown that, due to various aerodynamic effects, predictions solely based on initial position and velocity are largely inaccurate [12]. This circumstance makes the problem difficult and raises the question on how humans, such as trained baseball players, solve it, and which computational strategies are best-suited for solving it.

In this work, we are most interested in the computational aspect of the ball catching problem. We thus begin with a rigorous mathematical formalization of the problem and outline basic solution strategies later in this section. We present solution strategies brought forward by the generalist and the specialist camp: the generalist approaches are based on a *Cartesian representation* of the task, and we will therefore term them *Cartesian control strategies*. The specialist approaches are based on an *angular representation* of the task, leading to a set of *angular control strategies*.

### 2.1 Ball

We assume a ball with mass *m*, radius *r*, denote the three-dimensional ball position by **b** = [*b*_*x*_, *b*_*y*_, *b*_*z*_]^*T*^, its velocity by $\dot{b}$ and its acceleration by $\ddot{b}$. The dynamics of the ball trajectory in an ideal environment are parabolic and given by the following differential equation:

$$\begin{array}{c} \ddot{b}=(\begin{array}{c} 0\\ -g\\ 0 \end{array}), \end{array}$$ (1)

assuming given initial conditions for position **b**(0) and velocity $\dot{b}\left(0\right)$. In our formalization, the *y*-axis corresponds to the vertical direction (“up”) and *g* = 9.81 denotes the average magnitude of gravity on earth. We assume the ball is launched at *t* = 0 and denote the ball impact time by *t* = *T*, thus *b*_*y*_(0) = *b*_*y*_(*T*) = 0.

In a more realistic setting the parabolic flight trajectory is affected by drag [12]. The drag force acts on the ball in the opposite of its tangential velocity and induces the following dynamics:

$$\begin{array}{cc} \ddot{b} & =(\begin{array}{c} 0\\ -g\\ 0 \end{array})-\frac{1}{2}\rho c_{d}\frac{A}{m}{\dot{b}}^{2}, \end{array}$$ (2)

where *ρ* is the air density, *A* = *πr*^2^ the ball’s cross-section and *c*_*d*_ the drag coefficient which varies for different types of balls.

### 2.2 Agent and controller

We denote the agent by **a** = [*a*_*x*_, *a*_*z*_]^*T*^ and assume that it moves on the horizontal plane. We assume that we can directly control the agent’s acceleration using control input **u** = [*u*_*x*_, *u*_*z*_]^*T*^, and assume the agent’s absolute velocity and acceleration to be constrained by ${\dot{a}}_{\text{max}}$ and ${\ddot{a}}_{\text{max}}$:

$$\begin{array}{c} \ddot{a}=u. \end{array}$$ (3)

**Fig 1:**
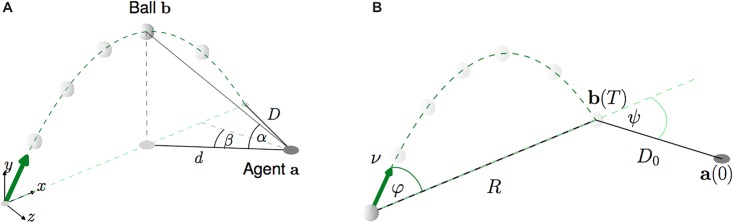
Illustration of the outfielder ball catching problem and all involved quantities. (A) Configuration at time *t*. World frame orientation depicted at the bottom left. (B) Initial configuration at time *t* = 0.

Additionally, we introduce notation to specify important relationships between agent and ball: the *ground distance* $d\left(t\right)=\sqrt{{\left(a_{x}\left(t\right)-b_{x}\left(t\right)\right)}^{2}+{\left(a_{z}\left(t\right)-b_{z}\left(t\right)\right)}^{2}}$ and *agent-to-impact distance* $D\left(t\right)=\sqrt{{\left(a_{x}\left(t\right)-b_{x}\left(T\right)\right)}^{2}+{\left(a_{z}\left(t\right)-b_{z}\left(T\right)\right)}^{2}}$, depicted in Fig 1A, and the *initial agent-to-impact distance* *D*(0) = *D*_0_, depicted in Fig 1B. Note that *d*(*t*) and *D*(*t*) vary over time whereas *D*_0_ is a fixed initial parameter.

#### 2.2.1 Cartesian representation

The agent and ball positions and velocities $a,\dot{a},b,\dot{b}$ correspond to the *Cartesian representation* of the ball catching scenario. It will form the basis of the *Cartesian control strategies*, the strategies brought forward by the generalist approach. These try to estimate $a,\dot{a},b,\dot{b}$ from the agent’s sensors and assume a *dynamic model* of ball and agent that closely matches the real dynamics given in Eqs (1), (2) and (3) to be available to the agent. In practice, it is not possible for the agent to get a perfect estimate of all quantities. In particular, the agent’s distance to the ball is difficult to estimate using monocular vision [13]. We will see later how these approaches perform with imperfect estimates or when their dynamic model does not match the real dynamics.

### 2.3 Task

The task of the agent is to arrive at the impact point of the ball at the same time as the ball while respecting the agent’s motion constraints:

$$\underset{a}{\text{minimize}}\;\|b\left(T\right)-{a\left(T\right)\|}^{2},$$ (4)

$$\text{subject}\;\text{to}\;\;\|\dot{a}\left(t\right)\|\leq {\dot{a}}_{\text{max}},$$ (5)

$$\|\ddot{a}\left(t\right)\|\leq {\ddot{a}}_{\text{max}}.$$ (6)

for 0 ≤ *t* ≤ *T*, where *T* denotes the ball’s impact time. We refer to Eq (4) as the *terminal distance* objective and denote it by $L_{\text{terminal}\;\text{distance}}$.

We will see later how the Cartesian and angular strategies approach this problem.

### 2.4 Vertical viewing angle

The specialist approaches do not attempt to estimate the agent and ball coordinates. Instead, they operate on two relative angles between agent and ball. The first one is the *vertical viewing angle* *α* between the ground plane and the ball, as seen from the agent’s perspective. It is shown in Fig 1A and is computed as:

$$\begin{array}{c} \alpha \left(t\right)=\arctan\frac{b_{y}\left(t\right)}{d(t)}, \end{array}$$ (7)

where *d*(*t*) denotes the ground distance defined above.

Of particular interest is the *tangent of the vertical viewing angle*:

$$\begin{array}{c} \theta \left(t\right)≜\tan\alpha \left(t\right)=\frac{b_{y}\left(t\right)}{d(t)}. \end{array}$$ (8)

An important property of *θ* is that it can be directly perceived using a monocular visual sensor, such as a camera; as we will see, angular control strategies only require an estimate of *θ* up to a constant scaling factor. This clearly distinguishes *θ* from the Cartesian representation which requires estimating the agent’s distance to the ball.

Some angular strategies require the estimation of derivatives of *θ*(*t*). We assume that these are computed using finite differences:

$$\begin{array}{c} \dot{\theta }\left(t\right)=\frac{\theta (t)-\theta (t-\Delta t)}{\Delta t},\;\ddot{\theta }\left(t\right)=\frac{\dot{\theta }\left(t\right)-\dot{\theta }\left(t-\Delta t\right)}{\Delta t}, \end{array}$$ (9)

given time step Δ*t* > 0.

### 2.5 Bearing angle

In addition to the vertical angle, we define the *bearing angle* *β*. It lies on the horizontal plane (see Fig 1A) and computes as the angle between two lines: a line connecting the agent and the ball’s projection on the ground plane *D*_*t*_; and an arbitrary exocentric (that means: defined in global frame) reference line, indicated by the gray dashed line in Fig 1A. To simplify notation, we assume that the exocentric reference line is always orthogonal to the ball’s flight trajectory. Then *β* can be computed from the difference in the agent’s and ball’s coordinates as follows:

$$\begin{array}{c} \beta \left(t\right)=\arctan\frac{a_{x}\left(t\right)-b_{x}\left(t\right)}{a_{z}\left(t\right)-b_{z}\left(t\right)}. \end{array}$$ (10)

Computing *β* from a camera image is a bit more difficult than computing *α*. The reason is that the agent needs to track its rotation to maintain the same exocentric reference line over time [14]. In this work, we assume that the agent does not rotate, and thus maintains a constant exocentric reference line and adjusts ${\hat{x}}_{\text{ref}}$ accordingly while moving.

#### 2.5.1 Angular representation

We refer to the pair *θ*, *β* as the *angular representation*, possibly replacing *θ*, *β* by higher derivatives, for example $\dot{\theta },\dot{\beta }$. In the two-dimensional case, the angular representation only consists of *θ* (or its derivatives). It will form the basis of the angular control strategies presented later.

### 2.6 Cartesian control strategies

We now have all the ingredients to define control strategies for solving the ball catching problem. We begin with the Cartesian strategy which is based on the Cartesian representation, consisting of the agent and ball positions and velocities.

The main idea of Cartesian controllers is to predict the impact point of the ball *R* and arrive at this point at the impact time (at the latest). Thus, these strategies are sometimes termed *trajectory prediction* strategies. Predicting the impact point is very easy if we have an accurate estimate of the current ball position and velocity (with respect to the agent) and if we know the ball dynamics, as we will show in Section 4.1.

The proposed Cartesian controller implementations mainly differ with respect to how they use the impact point estimate to guide the agent. A naive control strategy runs to the impact point as fast as possible [15]—but this is neither required nor helpful: first, the agent only has to arrive at the impact point at impact time. Second, the ball’s trajectory might be perturbed in midflight. Therefore, the agent is better off if it minimizes the risk of missing the ball in case it is affected by a perturbation.

A suitable approach to build such a risk-averse controller is the optimal control framework, for example linear-quadratic Gaussian control (LQG). We will describe in great detail how to apply LQG to the ball catching problem in Section 4.1. We will see that LQG is able to catch the ball in many cases, but its performance critically hinges on the ability to accurately predict the impact point.

An alternative optimal control approach for ball catching has been recently presented by [16]. It is based on the same Cartesian representation as LQG but uses a belief space planning method based on model-predictive control (*MPC*). MPC allows to deal with a more realistic agent model than the one considered here, but evaluating MPC in our simplified setting is possible because the agent model it assumes is strictly more general than the one we consider here. Our experiments will show that both LQG and MPC exhibit similar behavior in the simplified setting because they both rely on an accurate prediction of the impact point. We will see that an impaired ability of predicting the impact point equally affects all Cartesian controllers—including MPC.

### 2.7 Angular control strategies

An alternative set of strategies that does not require the prediction of the impact point is given by the angular control strategies. These are a bit less intuitive to understand than the Cartesian strategies, and various different implementations have been proposed in the literature. We focus here on the two most prominent types of the strategies: Chapman’s strategy and the Linear Optical Trajectory (LOT) strategy.

#### 2.7.1 Chapman’s strategy

**Fig 2:**
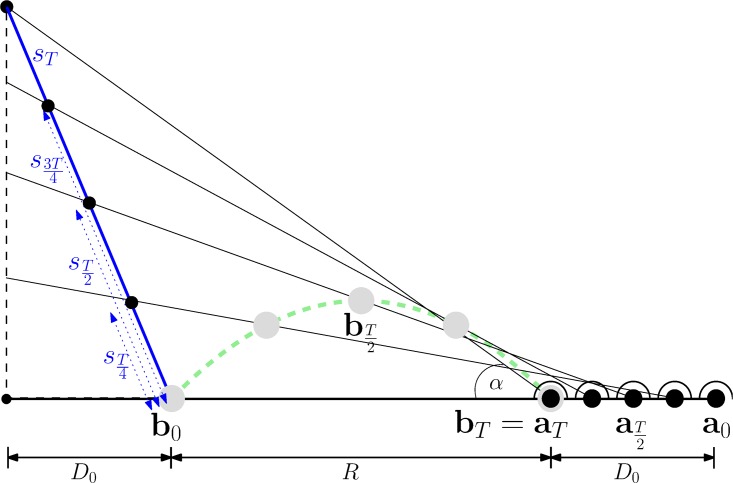
An intuitive explanation of Chapman’s strategy. (Adapted from [7]). It states that for an ideal parabolic ball trajectory and an agent moving towards the impact point with constant velocity $a_{x}\left(t\right)=-\frac{a_{0}-R}{T}$ the tangent of the vertical viewing angle *θ*(*t*) = tan *α*(*t*) rises with a constant rate. This is illustrated by the blue line segments whose length is linear both in *θ* since *s*_*t*_ = (*R* + *D*_0_)*θ*(*t*) and in time since *s*_*nt*_ = *ns*_*t*_; hence, *θ* is linear, too. We will show this formally in Section 4.2.

[5] presented an important finding that triggered a whole research field dedicated to angular control for projectile interception. In his paper, he presented what we call *Chapman’s strategy*: he was able to show that an agent arrives at the ball’s impact point exactly at impact time if the agent runs such that the tangent of the angle *θ* between ground plane and the ball rises at a constant rate. Moreover, he showed that in this case the agent’s velocity is constant. Fig 2 provides a visual explanation of this insight, and we prove it formally in Section 4.2.

To implement Chapman’s strategy, we need to address two issues. First, we need to find a control law that ensures that the agent runs such that *θ* rises linearly. Several controller implementations have been suggested, one of the most popular ones being *optical acceleration cancellation* (*OAC*, [17]). It implements Chapman’s strategy by tracking the acceleration of the tangent of the vertical viewing angle $\ddot{\theta }$ and adjusting the agent’s motion such that $\ddot{\theta }$ remains zero. We review OAC and other implementations of Chapman’s strategy in Section 4.2.

The second issue is that we need is how to control the lateral orientation of the agent. Chapman suggests to adjust the lateral movement such that the bearing angle remains constant, a strategy known as *constant bearing angle (CBA)*.

The obvious advantage of Chapman’s strategy is that it does not require an estimate of the impact point. However, the optimal-control based Cartesian controllers come with strong guarantees that reliably predict their behavior, whereas little theoretical knowledge about Chapman’s strategy is available. Hence, a main focus of our work is to provide a theoretical understanding of how and why Chapman’s strategy works. We additionally verify our findings in a large array of simulation experiments.

#### 2.7.2 Linear Optical Trajectory

Chapman’s strategy is not the only angular control strategy that has been suggested. The most important competitor is the *linear optical trajectory (LOT)* strategy [7]. Similarly to Chapman’s strategy, LOT uses the viewing and bearing angles to adjust the agent’s running trajectory. The main difference to Chapman’s strategy is that LOT does not command the vertical and the bearing angle independently, but adjusts the agent’s running speed such that the ball follows a linear trajectory from its viewpoint, which is equivalent to keeping $\frac{\frac{d}{dt}\tan\alpha \left(t\right)}{\frac{d}{dt}\tan\beta \left(t\right)}$ constant. This can result in a different running path, and it has been argued that it is more biologically plausible as it does not treat *θ* and *β* independently.

Although various studies for ball but also frisbee catching [18–20] support the LOT model, it is still debated whether LOT or Chapman’s strategy (or optimal control) account better for explaining human behavior [16, 21]. We do not attempt to answer whether one strategy resembles human behavior better than another. Instead, we aim to understand the relationship of generalist and specialist strategies for ball catching. We therefore focus on Chapman’s strategy (OAC with CBA) and use it as a representative of the specialist strategies for ball catching in the remainder of this paper.

## 3 Related work

In this section, we give a brief overview of the main arguments brought up in favor of the specialist and generalist views. Most of the discussions and studies regarding this controversy have addressed *single decision making*, i.e. *classification and regression* problems, whereas ball catching is a *sequential decision making*, i.e. *control* problem.

We begin by briefly reviewing arguments regarding single decision making, then turn to control problems in general and finally review related work regarding ball catching.

### 3.1 Single decision making: Classification and regression

In the generalist approach, single decision making problems are often tackled using supervised learning. Various theoretical results [22, 23] and practical examples [24] show that supervised learning is very successful at solving decision making problems—given that a sufficient amount of training data is available. Specialists argue that for highly unpredictable and uncertain environments with low amounts of data available, statistical learning is inferior to simple rules of thumb, “heuristics”. [10] define heuristics as “processes that ignore information and enable fast decisions”. He views the mind as an *adaptive toolbox* that employs various heuristics and applies them to cognitive tasks. Gigerenzer’s definition falls short of explaining how to find heuristics. He suggests that heuristics follow a certain pattern, such as a search rule, a decision rule and a stopping rule, but it is not clear how to use this pattern as a template for solving novel tasks, for example for *finding* rather than applying the ball catching heuristic. [4] defines heuristics in the context of search, where he considers a method to be heuristic if it only searches for solutions that are “good enough” but not necessarily optimal. He calls such a solution *satisficing*. Gigerenzer criticizes that Simon implies a accuracy–effort trade-off, assuming that more time and computation would result in a better and more optimal solution. Gigerenzer argues that this does not hold true and that heuristics can be perform better than the optimal solution, for example a statistical learner.

Indeed, statistical learning theory supports Gigerenzer’s argument. [25] showed that any statistical learner is prone to *overfitting* when the problem exhibits high uncertainty and lacks sufficient amounts of training data. Therefore, in such cases heuristics can outperform statistical learning [10, 11]. Statistical learning theory also explains when the opposite effect of *underfitting* occurs: a learner that relies on biases that are too strong or inadequate for the task make systematic mistakes [26].

To conclude, statistical learning theory perfectly explains heuristics as biased solutions that are optimal under uncertainty. We thus consider the controversy between generalists and specialists for single decision making as resolved. The main question then becomes what constitutes a suitable bias for a task and how task-general or task-specific such biases need to be.

### 3.2 Sequential decision making: Control

The sequential decision making setting still lacks a clear cut answer to the controversy between generalists and specialists. The reason is that the insights from the single decision making setting do not address all difficulties of the sequential setting. For example, the temporal coupling of decisions often renders supervised learning impractical as pure imitation of a known sequence of (even optimal) actions might lead to inferior performance [9]. This motivates approaches such as reinforcement learning [27].

Similar to single decision making, specialists advocate rules of thumb and heuristics to be the main driver for human decision making. However, these heuristics are usually task-specific. Therefore, we now discuss the differences between generalist and specialist approaches to decision making directly in the context of ball catching.

We now cover related work on Cartesian and angular control strategies for ball catching.

#### 3.2.1 Cartesian control strategies

As explained in Section 2.6, Cartesian control strategies are based on a prediction of the ball’s landing point, or more generally on the prediction of the ball trajectory. A large body of work in psychology advocates that prediction plays an important role for cognition [28] and particularly for motor control [28–30]. Experimental studies showing that prediction plays an important role in ball catching have been presented, too [31, 32]. In ball catching, a predictive strategy has the great advantage that it can effectively cope with cases where the ball goes out of the field of view [16]. In general, the main advantage of viewing cognition as predictive modeling is that it provides a generally applicable computational framework for explaining human behavior. Moreover, it can often be directly transferred to artificial systems. Hence, the trajectory prediction strategy is the method of choice in most robotic applications for ball catching [33, 34]. These applications, however, only study catching in closed-room environments and thus do not address difficulties faced in scenarios such as baseball: due to the long flight time the various aerodynamic forces such as drag or Magnus forces affect the ball trajectory significantly [12]. Therefore, it is not clear if the predictive approach to ball catching can be successfully applied in such scenarios.

#### 3.2.2 Angular control strategies

The view of equating cognition with predictive modeling is not undisputed. As mentioned above, [10] advocates that humans rely on simple heuristics and considers Chapman’s strategy as an instance of this heuristic approach. The simplicity of Chapman’s strategy has triggered a large body of work in psychology and engineering to study whether humans use this strategy and how it could be implemented [6, 35–37]. Most of them study the setting presented in Section 2, but extensions that incorporate human constraints have been suggested, too [8].

Several studies investigate implementations of Chapman’s strategy and their performance under different conditions, mostly in physical simulation experiments. One of the earliest studies [38] notices the effect of air resistance on the ball trajectory, which Chapman neglected. However, the author wrongly concludes that Chapman’s strategy is not applicable in the case of air resistance [39] and does not study the influence of additional perturbations. [40] suggests different control architectures for implementing Chapman’s strategy and tests them in two- and three-dimensional simulations. He reports that Chapman’s strategy is more robust when implemented with bang-bang, rather than proportional control, especially when modeling human constraints such as sensorimotor delays, velocity and acceleration constraints. Except for air resistance, he does not consider perturbations on the ball trajectory, though. [41] suggests a different variant of Chapman’s strategy which controls the velocity rather than the acceleration of the tangent, the *control of optical velocity (COV*) strategy. He evaluates it in a set of simulated experiments with random noise and wind perturbations, with the main goal of showing its superiority to LOT. We will derive a mathematical relationship between COV and OAC in Section 4.3 and propose a method that combines the strengths of both approaches. [42] implement OAC on a simulated mobile robot with differential drive, focusing on deriving the full (image) Jacobians required to map image errors to wheel motor commands. They only evaluate their system in two exemplary experiments. [43] study the performance of a bang-bang OAC model for a variety of different initial configurations of the agent and various perturbations, but they neglect air resistance.

Finally, simplified variants of Chapman’s strategy have been implemented in real-world robotic experiments. [44] test different variants of Chapman’s strategies and suggest to preferably use bang-bang, following a similar line of reasoning as [40]. [45] propose a system for ground ball interception but use an *angle prediction* strategy (AP) rather than OAC: the error is computed as the difference of the current and a linear prediction of the tangent of the angle in the next time step. We will discuss this strategy in Section 4.3 and show that it is equivalent to the COV strategy. Finally, [39] implement the OAC strategy on a mobile robot and study the behavior when catching a balloon. They also report that active servoing of the camera resulted in more stable behavior than using a passive camera.

## 4 A comparison of Cartesian and angular control for ball catching

The goal of this section is to systematically compare the Cartesian and angular control approaches presented in the previous section. We show that angular control strategies, brought forward by the specialist view, generalize over systematic perturbations of the ball trajectory, such as air resistance, whereas generalist strategies based on the Cartesian representation generalize better over perturbations induced by Gaussian noise.

We begin by formalizing and implementing these strategies, amongst them novel strategies not considered in the literature. Our treatment includes an in-depth theoretical analysis of all presented strategies as well as an empirical study in terms of extensive physical simulation experiments.

### 4.1 Cartesian control strategies

In Section 2.6, we discussed different ways to implement a Cartesian controllers for ball catching. We now formalize a representative for the Cartesian control strategies by instantiating the *linear-quadratic Gaussian control framework (LQG)*. In a nutshell, applying LQG to a control problem consists of two phases: in the first, *offline* phase, LQG uses known dynamic and observation models (of agent and ball) and a cost function (rewarding catching success) to compute the control function **u** = *f*_*t*_(**z**), which computes optimal control outputs **u** for every possible observation **z** (position of ball and agent) of the underlying state **x** (position and velocity of ball and agent). The subscript *t* indicates that this function is time-varying, that means, it depends on the *time-to-impact* (time until the ball hits the ground). In the second, *online* phase, at every time step *t* the agent makes an observation **z**_*t*_, infers the most likely state **x**_*t*_, estimates the time-to-impact *T* and applies *f*_*t*_ to compute **u**.

Note that whereas standard LQG requires the dynamic and observation models to be linear and the cost function to be quadratic, nonlinear extensions such as *iterative LQG (iLQG)* exist [46]. For a more in-depth treatment on optimal control see [47] and the supplementary material (S4 Text).

In order to apply LQG to our problem, we now define (1) a *state representation*, (2) a *(locally) linear dynamics model*, (3) an *observation* representation as well as an *observation model*, (4) a *finite-horizon cost function* and (5) a way to measure the *time-to-impact*.

#### 4.1.1 State, observation and dynamics

The formalization of the ball catching problem (Sections 2.1 and 2.2) directly provides the state and dynamics required to instantiate the LQG framework. LQG requires the state representation **x** to be *Markov*: **x**_*t*_ must fully define the system’s state at time *t* such that adding more information about the past of **x**_*t*_ cannot improve the prediction of any future state. A suitable state representation fulfilling this criterion is the Cartesian representation presented in Section 2.2. Section 2.1 also presented the dynamics model governing ball and agent motion based on this state. The dynamics are linear in the ideal case and locally linear when assuming drag. Moreover, LQG allows us to relax the assumption that we can perfectly estimate all quantities of the Cartesian representation. Instead we assume the agent’s observation of these quantities to be perturbed by Gaussian noise.

We thus define the state representation **x** and the observation **z** as

$$\begin{array}{cc} x & ={\left[b_{x},{\dot{b}}_{x},b_{y},{\dot{b}}_{y},b_{z},{\dot{b}}_{z},a_{x},{\ddot{a}}_{x},a_{z},{\ddot{a}}_{z}\right]}^{T}, \end{array}$$ (11)

$$\begin{array}{cc} z & ={\left[{\tilde{b}}_{x},{\tilde{b}}_{y},{\tilde{b}}_{z},{\tilde{a}}_{x},{\tilde{a}}_{z}\right]}^{T}. \end{array}$$ (12)

To formalize the state dynamics for LQG, we use the ball dynamics stated in Section 2.1 and bring them into the linear form required by LQG. Similarly, we need to define a linear observation model mapping states to observations. All details on how to adapt the state dynamics and observation model for the LQG framework are provided in the supplementary materials (S2 Text).

Remark: in our formalization, the Cartesian representation defines agent and ball position with respect to a global reference frame, whereas the angular representation of the ball is defined relative to the agent. However, this is only a matter of convenience because separating agent **a** and ball **b** makes the equations more comprehensible. We could easily define the ball in a relative reference frame $\hat{b}=b-a$, omit the agent from the state and obtain equivalent dynamics.

#### 4.1.2 Cost function

Section 2.3 formalized the ball catching task as the minimization of the distance between agent and ball at impact time while respecting the agent’s motion constraints (Eqs 4–6):

$$\underset{a}{\text{minimize}}\;L_{\text{terminal}\;\text{distance}},$$ (13)

$$\text{subject}\;\text{to}\;\;\|\dot{a}\left(t\right)\|\leq {\dot{a}}_{\text{max}},$$ (14)

$$\|\ddot{a}\left(t\right)\|\leq {\ddot{a}}_{\text{max}}.$$ (15)

where $L_{\text{terminal}\;\text{distance}}=\|b\left(T\right)-a\left(T\right){\|}^{2}$ denotes the *terminal distance* cost. The main objective $L_{\text{terminal}\;\text{distance}}$ is a finite-horizon term and thus suited for LQG, but LQG cannot directly incorporate the hard agent motion constraints. Instead, LQG requires a single cost function in quadratic form. To address these requirements, we define the cost for LQG as a sum of $L_{\text{terminal}\;\text{distance}}$ and an additional regularization term that penalizes *control effort*,

$$\begin{array}{cc} L & =w_{\text{dist}}L_{\text{terminal}\;\text{distance}}+w_{\text{ctrl}}L_{\text{control}\;\text{effort}} \end{array}$$ (16)

$$\begin{array}{cc} L_{\text{control}\;\text{effort}} & =\sum_{t=0}^{T}{\left\|u\left(t\right)\right\|}^{2}, \end{array}$$ (17)

where the hyperparameters *w*_dist_ and *w*_ctrl_ balance the influence of the two cost terms.

Note that omitting $L_{\text{control}\;\text{effort}}$ would result in a controller that tries to move the agent directly to the impact point within a single time-step—a motion that is impossible in the general case given the agent’s motion constraints.

#### 4.1.3 Computing time-to-impact

Finally, LQG requires a way to estimate the current time horizon. In the ball catching scenario, this corresponds to the time-to-impact *T*. For the ideal case, we can compute it directly solving the ball dynamics equation (Section 2.1) for *b*_*y*_(*t*) = 0. We thus obtain,

$$\begin{array}{cc} -0.5gT^{2}+{\dot{b}}_{y}\left(t\right)T+b_{y}\left(t\right) & =0, \end{array}$$ (18)

and solve for *T*, assuming the current ball position *b*_*y*_(*t*) and velocity ${\dot{b}}_{y}\left(t\right)$ to be known.

In the presence of drag, no closed-form solution of the dynamics is known. In this case, we compute *T* by running a forward simulation of the ball trajectory using the current estimate of **x** and the (drag) dynamics model.

#### 4.1.4 Controller analysis

We are interested in the performance of LQG both in the ideal case and in the presence of perturbations. We consider two different types of perturbations.

**Random perturbations**: It is by design that LQG is robust to Gaussian noise both in the dynamics as well as the observation [47]. This suggests that LQG is robust to any form of Gaussian perturbations.

**Systematic perturbations**: To get an intuition for how LQG is affected by systematic perturbations, we look at how it is influenced by drag. Since we know that the drag induces different ball dynamics than the ideal case we inspect the optimal control gains obtained when assuming a model either with or without drag.

We begin by stating the insights that hold irrespective of whether we assume drag or not. First, we observe that LQG computes separate, identical gains for *u*_*x*_ and for *u*_*z*_; this makes sense for the ball catching problem as the two coordinates are decoupled [30]. Second, we see that, for both models, the gains can be decomposed into a constant, proportional and derivative term which vary over time *k*_*p*_(*t*), *k*_*d*_(*t*) and *k*_*c*_(*t*). Therefore, we see that LQG learns a (biased) PD-controller of the following form

$$\begin{array}{c} u_{x}=k_{p}\left(x_{b}-x_{a}\right)+k_{d}\left({\dot{x}}_{b}-{\dot{x}}_{a}\right)+k_{c}, \end{array}$$ (19)

where we have omitted the time indices to improve readability (*u*_*z*_ can be defined in an analogous way).

**Fig 3:**
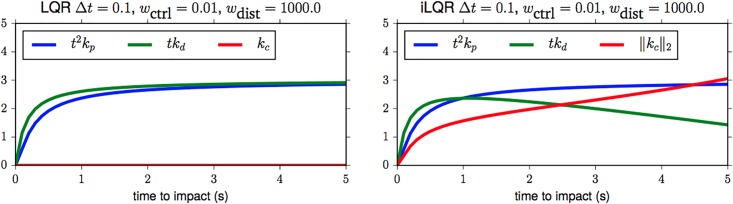
Analysis of the control gains computed for the ideal case and for drag. (A) LQR *without* drag. (B) iLQR *with* drag.

We can now analyze how these gains differ depending on whether the model assumes drag or not. Fig 3 shows the gains in relation to the time to impact. We see that *k*_*p*_ and *k*_*d*_ are the dominating terms for LQR, and that *k*_*p*_ has a (reciprocal) quadratic and *k*_*d*_ a linear dependence on *t*. This reflects the quadratic form of the idealized ball trajectory equation. When the model incorporates drag (Fig 3B), the influence of the constant term *k*_*c*_ increases with the time to impact. This correlates with the fact that with increased flight time the influence of drag increases, too. Due to the non-linear nature of the drag force no linear control term can account for it, and different drag parameters (mass, radius, drag coefficient) thus affect this term and the behavior of the controller.

We thus hypothesize that wrong model assumptions induces a systematic error in LQG and degrade the performance of LQG for unmodeled systematic perturbations.

### 4.2 Angular control strategies

In this section, we turn to a formalization and theoretical analysis of the angular control strategies for ball catching. We focus our analysis on the simplified two-dimensional case where the ball moves in the plane and the agent on a line (*b*_*z*_ = *a*_*z*_ = 0). It is based on *Chapman’s strategy*, described in Section 2.7.1. We provide theoretical results that make predictions how and under which conditions this strategy is successful at the ball catching task (Section 4.2), derive different control laws for implementing it, and then provide a discussion of whether the angular representation qualifies as a state representation and close by extending the strategy to the full three-dimensional case (Section 4.3).

We begin our analysis by stating why the angular strategies are expected to result in successful agent motion. To that end, we present a reformulation of Chapman’s proof [5], showing that, under the assumption of a perfect parabolic trajectory, Chapman’s strategy leads to successful interception of the ball. We extend Chapman’s proof and give additional important insights about the angular representation, which allows us to design novel robust control strategies that implement Chapman’s strategy in the next section.

All of the following proofs were verified using *SymPy* [48], and the corresponding code has been made publicly available at https://github.com/shoefer/ball_catching.

Our proofs concentrate on the two-dimensional case. To facilitate notation for this case, we rewrite the equations of motions as follows.

**Ball Trajectory**: We begin by expressing the ball dynamics as a function of time *t*:

$$b_{x}\left(t\right)=\nu t\cos\varphi +b_{x,0},$$ (20)

$$b_{y}\left(t\right)=\nu t\sin\varphi -\frac{1}{2}gt^{2}+y_{b,0},$$ (21)

where *g* = 9.81 denotes gravity (on earth). $\nu =\sqrt{{\dot{b}}_{x,0}^{2}+{\dot{b}}_{y,0}^{2}}$ and $\varphi =\tan^{-1}\frac{{\dot{b}}_{y,0}}{{\dot{b}}_{x,0}}$ represent the initial velocity of the ball in polar, and ${\dot{b}}_{x,0}$ and ${\dot{b}}_{y,0}$ in Cartesian coordinates. Without loss of generality, we assume *b*_*x*_(0) = *b*_*x*,0_ = 0 and *b*_*y*_(0) = *b*_*y*,0_ = 0.

**Impact Time and Impact Point**: By setting (Eq 21) to zero and solving for t, we obtain the ball’s *impact time* *T*. We obtain the range or *impact point* *R* by resubstituting *T* into (Eq 20):

$$\begin{array}{c} T=\frac{2\nu }{g}\sin\varphi \end{array}$$ (22)

$$\begin{array}{c} R=\frac{\nu^{2}}{g}\sin2\varphi \end{array}$$ (23)

**Agent Trajectory**: In the two-dimensional case we assume *a*_*z*_(*t*) = 0 for all *t*. The agent thus moves on a line and we write *a*(*t*) = *a*_*x*_(*t*). We further assume that the agent’s initial position *a*(0) = *a*_*x*,0_ does not coincide with the ball’s initial position, *a*_*x*,0_ ≠ *b*_*x*,0_.

**Agent Reference**: The following results neglect the agent’s velocity and acceleration constraints. Instead, they only deal with the *desired* agent motion *a*_ref_(*t*), which we distinguish from the actual agent motion *a*(*t*). We also refer to *a*_ref_(*t*) as the *agent reference*. Control laws relating *a*_ref_ and *a* are covered in the next section.

**Chapman’s Proof**: From (Eq 8) we know that the tangent of the vertical viewing angle *θ* is a function of the agent and ball position. Chapman’s strategy assumes that the velocity of the vertical viewing angle $\dot{\theta }$ is constant. We can express this assumption with the following equation:

$$\begin{array}{cc} \frac{b_{y}\left(t\right)}{a_{\text{ref}}\left(t\right)-b_{x}\left(t\right)} & ={\dot{\theta }}_{\text{ref}}\;t+{\dot{\theta }}_{\text{ref},0}\\ & ={\dot{\theta }}_{\text{ref}}\;t+\frac{b_{y,0}}{a_{0}}\\ & ={\dot{\theta }}_{\text{ref}}\;t, \end{array}$$ (24)

where ${\dot{\theta }}_{\text{ref}}$ is a constant representing the *reference velocity of the tangent of the vertical viewing angle*, called *tangent reference velocity*. ${\dot{\theta }}_{\text{ref},0}=\frac{b_{y,0}}{a_{0}-b_{x,0}}$ is the initial tangent reference velocity at time *t*, which is zero as we assume *b*_*y*_(0) = *b*_*y*,0_ = 0 (and *b*_*x*,0_ ≠ *a*_0_). Moreover, we assume *a*_ref_(0) = *a*(0) = *a*_0_

Given these equations, we now prove the following statement:

**Theorem 4.2.1.** (Chapman’s Proof). *Given a parabolic ball trajectory, parametrized by initial velocity ν* and launching angle *φ, the agent reference* *a*_ref_(*t*) *coincides with the ball position at time* *T*, *a*_ref_(*T*) = *R* = *b*_*x*_(*T*) *(“the agent intercepts the ball”) if the agent reference is chosen such that the tangent of the vertical viewing angle increases linearly,* $\dot{\theta }\left(t\right)=\text{const}$. [5].

*Proof*. By bringing all terms in (24) to the right-hand side and solving for *a*_ref_(*t*) we obtain the following expression:

$$\begin{array}{c} a_{\text{ref}}\left(t\right)=\frac{1}{{\dot{\theta }}_{\text{ref}}}(\nu t{\dot{\theta }}_{\text{ref}}\cos\varphi +\nu \sin\varphi -\frac{1}{2}gt) \end{array}$$ (25)

We now assess how far the agent is away from impact point *R* as we approach the impact time *T*, by computing the limit of *a*_ref_(*T* − *δ*) − *R* for *δ* → 0. After some modest algebraic manipulations we obtain:

$$\begin{array}{c} \lim_{\delta \to 0}a_{\text{ref}}\left(T-\delta \right)-R=\delta (-\nu \cos\varphi +\frac{g}{2{\dot{\theta }}_{\text{ref}}})=0. \end{array}$$ (26)

This limit is 0 since *δ* → 0 is multiplied with a constant expression.

We have now verified Chapman’s proof [5], proving the suitability of his strategy to intercept balls with parabolic trajectories. Next, we can prove a set of interesting lemmas regarding the relationship of the tangent reference velocity ${\dot{\theta }}_{\text{ref}}$ and the agent reference *a*_ref_.

**Lemma 4.2.2.** *For an agent that moves such that* Eq (24) *is satisfied, for every set of initial conditions a*_0_, *ν*, *φ there exists a unique consistent tangent reference velocity* ${\dot{\theta }}_{\text{ref}}^{*}$:

$$\begin{array}{c} {\dot{\theta }}_{\text{ref}}^{*}=\frac{\nu }{a_{0}}\sin\varphi . \end{array}$$ (27)

*Proof*. Evaluate Eq (25) for *t* = 0 and solve for ${\dot{\theta }}_{\text{ref}}$ (assuming *a*_ref_(0) = *a*_0_).

This lemma illustrates the tight connection between *a*_0_ and ${\dot{\theta }}_{\text{ref}}$. The next lemma, which has also been shown by [5, Eq 7], studies connection of ${\dot{\theta }}_{\text{ref}}$ and the agent reference *a*_ref_.

**Lemma 4.2.3.** *The agent reference that satisfies* Eq (24) *is constant, a*_ref_ = const*: the agent moves from its initial position a*_0_ *to the impact point R with constant velocity* [5].

*Proof*. By substituting ${\dot{\theta }}_{\text{ref}}$ in Eq (25) by ${\dot{\theta }}_{\text{ref}}^{*}$ from Lemma 4.2.2 we obtain the *consistent agent reference* $a_{\text{ref}}^{*}$:

$$\begin{array}{c} a_{\text{ref}}^{*}\left(t\right)=\frac{a_{0}}{\nu \sin(\varphi )}(\frac{\nu^{2}t}{a_{0}}\sin(\varphi )\cos(\varphi )+\nu \sin(\varphi )-\frac{1}{2}gt). \end{array}$$ (28)

By computing the derivative with respect to *t* we obtain:

$$\begin{array}{c} \frac{da_{\text{ref}}^{*}\left(t\right)}{dt}=\nu \cos\varphi -\frac{a_{0}g}{2\nu \sin\varphi }={\dot{a}}_{\text{ref}}^{*}, \end{array}$$ (29)

which is constant. Finally, it is easy to see that ${\dot{a}}_{\text{ref}}^{*}=\frac{R-a0}{T}$.

Lemmas 4.2.2 and 4.2.3 are constructive for creating an open-loop controller that implements Chapman’s strategy: the agent should estimate the consistent tangent reference velocity ${\dot{\theta }}_{\text{ref}}^{*}$ and run such that $\dot{\theta }\left(t\right)={\dot{\theta }}_{\text{ref}}$. It then runs with constant velocity ${\dot{a}}_{\text{ref}}^{*}$ towards the impact point. We will use this idea to suggest control laws for Chapman’s strategy in the next section.

**Fig 4:**
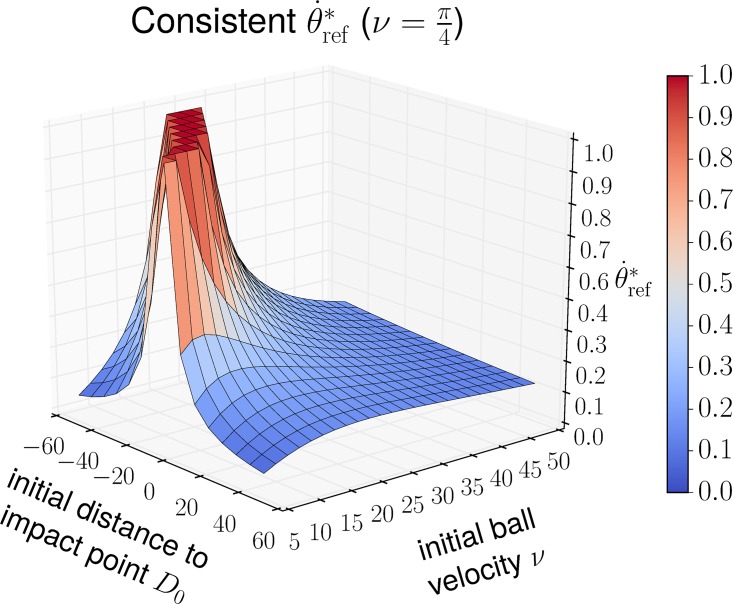
Consistent ${\dot{\theta }}_{\text{ref}}^{*}$ for varying initial distance from impact point *D*_0_ = *a*_0_ − *R* and varying initial ball velocity *ν* while keeping $\varphi =\frac{\pi }{4}$ fixed. Values greater 1 are clipped. Different values for *φ* only moderately scale the value of ${\dot{\theta }}_{\text{ref}}^{*}$ (see Eq 27).

In practice, however, the agent does not know the consistent tangent reference velocity ${\dot{\theta }}_{\text{ref}}^{*}$ because it does not have access to the initial conditions *a*_0_, *ν* and *φ*. To get an intuition about ${\dot{\theta }}_{\text{ref}}^{*}$, we first plot its value using Eq (27) for different initial conditions (Fig 4).

We see that ${\dot{\theta }}_{\text{ref}}^{*}$ has similar values for a wide variety of initial conditions. However, the following corollary shows that choosing an *inconsistent* tangent reference velocity can result in an agent reference *a*_ref_ that leads to undesired behavior.

**Corrolary 4.2.4.** *Given initial conditions a*_0_, *ν* *and φ, and the consistent tangent reference velocity* ${\dot{\theta }}_{\text{ref}}^{*}$ *resulting from* Eq (27). *If we use an inconsistent tangent reference velocity* ${\dot{\theta }}_{\text{ref}}^{∼}\neq {\dot{\theta }}_{\text{ref}}^{*}$ *for computing the agent reference a*_ref_, *the agent first moves to* $a_{0}^{∼}$, *which is the starting point for which* ${\dot{\theta }}_{\text{ref}}^{∼}$ *would be the consistent tangent reference velocity, before moving to the impact point R*.

**Fig 5:**
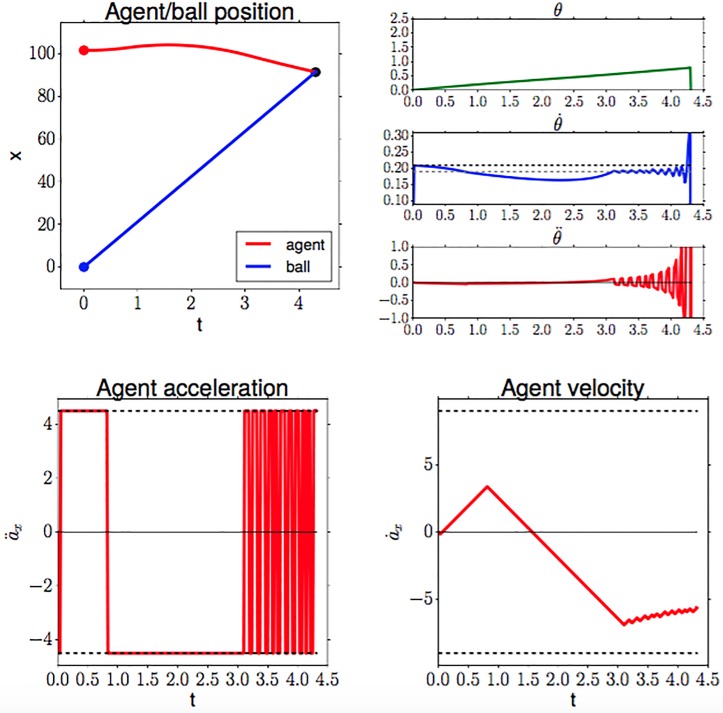
Chapman’s strategy for initial conditions $D_{0}=10m,\nu =30\frac{m}{s}$, without perturbations, and for an adversarially chosen tangent reference velocity ${\dot{\theta }}_{\text{ref}}^{∼}=0.1885$ (deviating by −0.02 from the consistent ${\dot{\theta }}_{\text{ref}}^{*}=0.2085$). The agent implements the COV strategy which is described in Section 4.3, using the simulation setup from Section 4.4. We see that the agent initially runs in the wrong direction, *away* from the ball, before turning at *t* = 0.8 to head towards the ball. While this deviation still allows the agent to catch the ball, ${\dot{\theta }}_{\text{ref}}^{∼}=0.161$ (deviation of −0.027 from ${\dot{\theta }}_{\text{ref}}^{*}$) would already result in an unsuccessful catch. Top left: agent and ball position. Top right: tangent of the vertical viewing angle and its derivatives; the dotted black line indicates the consistent ${\dot{\theta }}_{\text{ref}}^{*}$ value, the dotted gray line indicates the adversarially chosen inconsistent ${\dot{\theta }}_{\text{ref}}^{∼}$. Bottom left: agent velocity (dashed lines denote velocity contraints). Bottom right: agent acceleration (dashed lines denote acceleration constraints).

This corollary shows that we have to carefully choose the tangent reference velocity because every inconsistent tangent reference velocity ${\dot{\theta }}_{\text{ref}}^{∼}$ is linked to an initial position $a_{0}^{∼}$—an $a_{0}^{∼}$ that can lie in the *opposite direction* of the impact point *R*, even for very small deviations $|{\dot{\theta }}_{\text{ref}}^{*}-{\dot{\theta }}_{\text{ref}}^{∼}|$. Such an example is illustrated in Fig 5.

**Estimating Consistent Reference Velocity**: The obvious next question is how to estimate ${\dot{\theta }}_{\text{ref}}$ in the best way without knowing the initial conditions *φ*, *ν* and *a*_0_. One idea is to observe the tangent velocity shortly after the ball is launched, at some small $\tilde{t}>0$, and set ${\dot{\theta }}_{\text{ref}}^{∼}=\dot{\theta }\left(\tilde{t}\right)$ (in Section 4.3.4 we will introduce this strategy as *COV-IO*). To assess how severely this (inconsistent) choice of ${\dot{\theta }}_{\text{ref}}^{∼}$ deviates from the consistent ${\dot{\theta }}_{\text{ref}}^{*}$ and how it affects the agent reference, we compute how much the resulting inconsistent initial agent position $a_{0}^{∼}$ differs from the actual initial position *a*_0_.

**Lemma 4.2.5.** *Assume the initial starting position of the agent is a*_0_ = *R* + *D*_0_, *where D*_0_ *denotes the agent’s initial distance to the impact point R. If the agent observes* ${\dot{\theta }}_{\text{ref}}^{∼}=\dot{\theta }\left(\tilde{t}\right)$ *after* $\tilde{t}=\delta \cdot T$ *time steps*, 0 < *δ* < 1, *(that means after* 100 *⋅ δ percent of the entire trajectory length) the difference of the inconsistent initial agent position* $a_{0}^{∼}$ *differs from the actual initial position a*_0_ *by*

$$\begin{array}{c} a_{0}^{∼}-a_{0}=D_{0}\frac{\delta }{1-\delta }. \end{array}$$ (30)

*Proof*. We first compute ${\dot{\theta }}_{\text{ref}}^{∼}$ by solving Eq (24) for ${\dot{\theta }}_{\text{ref}}$, substituting *t* by $\tilde{t}=\delta \cdot T$ and substituting *a*_0_ by *R* + *D*_0_:

$$\begin{array}{c} {\dot{\theta }}_{\text{ref}}^{∼}=-\frac{\nu g(\delta -1)\sin(\varphi )}{D_{0}g-\delta \nu^{2}\sin(2\varphi )+\nu^{2}\sin(2\varphi )}. \end{array}$$ (31)

Next, we compute the inconsistent $a_{0}^{∼}$ by replacing ${\dot{\theta }}_{\text{ref}}$ in Eq (27) with ${\dot{\theta }}_{\text{ref}}^{∼}$:

$$\begin{array}{c} a_{0}^{∼}=\frac{1}{g(\delta -1)}(-D_{0}g+\delta \nu^{2}\sin(2\varphi )-\nu^{2}\sin(2\varphi )), \end{array}$$ (32)

and finally compute $a_{0}^{∼}-a_{0}=a_{0}^{∼}-R-D_{0}$ which yields Eq (30).

**Fig 6:**
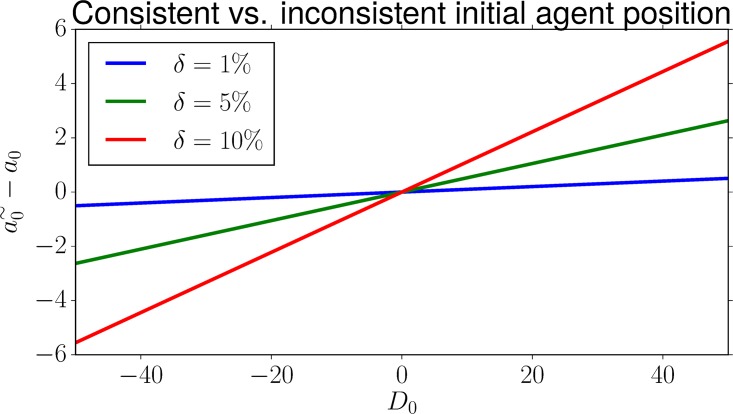
Difference between the inconsistent and the actual initial agent position, when observing the tangent reference velocity after 1%, 5% or 10% of the entire trajectory length.

Interestingly, the expression in Eq (30) only depends on the initial distance *D*_0_ of the agent to the impact point and the time the agent waits to observe the current reference velocity, *T* ⋅ *δ*. Fig 6 plots the error for different distances and observation times. We see that the error increases linearly with distance *D*_0_, and is lower the earlier the agent observes it.

**Perturbed Trajectories**: All results so far only hold for the ideal case, that is for a ball moving on an ideal parabolic trajectory. However, in realistic settings, the trajectory is perturbed by both random and systematic noise, such as drag. We now show that Chapman’s strategy adapts the agent reference to such noise:

**Lemma 4.2.6.** *If the ball trajectory is perturbed by some function ε*(*t*) *in the horizontal component*, ${b_{x}}^{\prime }\left(t\right)=b_{x}\left(t\right)+\varepsilon \left(t\right)$, *the agent reference adapts by changing to*

$$\begin{array}{c} a_{\text{ref}}^{\prime }\left(t\right)=\frac{1}{{\dot{\theta }}_{\text{ref}}}[\nu \sin\varphi -\frac{1}{2}gt+{\dot{\theta }}_{\text{ref}}(\nu t\cos\varphi +\varepsilon (t))]. \end{array}$$ (33)

*Proof*. It can be easily shown by substituting *b*_*x*_(*t*) in Eq (25) by *b*_*x*_(*t*) + *ε*(*t*).

**Fig 7:**
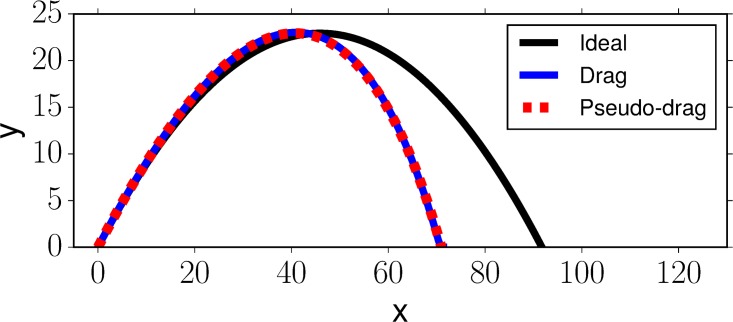
Example illustrating that drag can be closely approximated by the horizontal perturbation function *ε*(*t*). In this example, we simulate a ball with mass *m*_*b*_ = 0.15 kg, radius *r*_*b*_ = 0.0366 m, drag parameters *c*_*d*_ = 0.5, *A* = *πr*^2^, *ρ* = 1.293 and initial conditions $\nu =30\frac{\;m}{\;s}$, $\varphi =\frac{\pi }{4}$. The figure shows the ideal trajectory of the ball without drag (black line), with drag (blue line) and the fitted trajectory resulting by adding *ε*(*t*) = −1.11*t*^2^ to *x*_*b*_(*t*) (red dashed line). Note that *ε*(*t*) is different for every set of drag parameters and initial conditions.

Note that *ε*(*t*) can be in principle *any* source of noise. For example, we can approximate drag by *ε*(*t*), as exemplified in Fig 7. This finding shows why Chapman’s strategy has been previously reported to be robust to drag [38], and it clearly distinguishes it from the Cartesian control strategies which were not invariant to different drag parameters (Section 4.1.4). However, this result only characterizes the influence of noise on the agent *reference* trajectory; velocity and acceleration constraints influence the agent’s ability to follow the reference trajectory, and we will see in Section 4.4 that high-frequency perturbations causes problems in practical applications.

### 4.3 Control laws for Chapman’s strategy

The previous section has shown under which conditions Chapman’s strategy generates an agent reference trajectory *a*_ref_ that, if followed, guarantees interception of the ball. However, it ignored motion constraints that apply in realistic settings. We now discuss control laws for tracking *a*_ref_. In the related work section (Section 3), we already discussed three control laws for Chapman’s strategy: the *angle prediction (AP)* strategy directly uses the tangent *θ*, the *constant optical velocity (COV)* uses its velocity $\dot{\theta }$, and the *optical acceleration cancellation (OAC)* uses the acceleration $\ddot{\theta }$.

We now show that AP, COV and OAC all work equivalently, but differ with respect to how they estimate the tangent reference velocity ${\dot{\theta }}_{\text{ref}}$. We begin by formalizing the control laws that implement AP, COV and OAC. We then show how to relate these control laws to each other in order to demonstrate their equivalence.

To facilitate our analysis, we assume that the agent applies a simple proportional control law:

$$\begin{array}{c} u=k_{p}\;e, \end{array}$$ (34)

with some proportional gain *k*_*p*_ and error *e*. We can thus focus on how AP, COV and OAC define the control error *e*.

Remark: In Section 3 we mentioned that in practical implementations *bang-bang* control is used instead of P-control. This is due to the high system noise when sensing the vertical viewing angle and its tangent, respectively [40, 44] However, P-control and bang-bang control are equivalent if we set *k*_*p*_ to an extremely large value and assume that the control output *u* is clipped.

**Angle Prediction (AP)**: Assuming the tangent reference velocity ${\dot{\theta }}_{\text{ref}}$ to be known, the agent predicts the next value *θ* and compares it to the observed value:

$$\begin{array}{c} e_{\text{AP}}\left(t\right)=\left(\theta \left(t-\Delta t\right)+{\dot{\theta }}_{\text{ref}}\;\Delta t\right)-\theta \left(t\right). \end{array}$$ (35)

A variant of AP with explicit dependency on time *t* has also been suggested [49]:

$$e_{\text{AP}}^{\prime }\left(t\right)={\dot{\theta }}_{\text{ref}}\;t-\theta \left(t\right).$$ (36)

We see that equivalence $e_{\text{AP}}\left(t\right)=e_{\text{AP}}^{\prime }\left(t\right)$ holds if $\dot{\theta }\left(t\right)=\dot{\theta }\left(t-\Delta t\right)={\dot{\theta }}_{\text{ref}}$ for all *t*:

$$e_{\text{AP}}^{\prime }\left(t\right)={\dot{\theta }}_{\text{ref}}\;t-\theta \left(t\right)=\left(\theta \left(t-\Delta t\right)+\dot{\theta }\left(t-\Delta t\right)\;\Delta t\right)-\theta \left(t\right){=}_{\left(\dot{\theta }\left(t-\Delta t\right)\right)={\dot{\theta }}_{\text{ref}}}e_{\text{AP}}\left(t\right).$$ (37)

However, in practice $\dot{\theta }\left(t\right)$ is not constant, and thus we focus on *e*_AP_.

**Constant Optical Velocity (COV)**: COV [41] also assumes ${\dot{\theta }}_{\text{ref}}$ to be known and compares it to the current tangent velocity $\dot{\theta }$:

$$\begin{array}{c} e_{\text{COV}}\left(t\right)={\dot{\theta }}_{\text{ref}}-\dot{\theta }\left(t\right). \end{array}$$ (38)

**Optical Acceleration Cancellation (OAC)**: OAC does not assume ${\dot{\theta }}_{\text{ref}}$ to be known and instead zeros the acceleration $\ddot{\theta }$ [40, 43]:

$$\begin{array}{c} e_{\text{OAC}}\left(t\right)=-\ddot{\theta }\left(t\right) \end{array}$$ (39)

**AP, COV and OAC are equivalent**: We show how the previously proposed control laws are related through the error terms *e*_AP_, *e*_COV_ and *e*_OAC_.

**Theorem 4.3.1.** *AP, COV and OAC all maintain the tangent velocity of the vertical viewing angle equal to some desired reference velocity* ${\dot{\theta }}_{\text{ref}}$. *The different implementations of Chapman’s strategy only differ in the way this reference velocity is estimated*.

**Lemma 4.3.2.** *e_AP_ and e_COV_ only differ by a multiplication with a scalar, namely the frame rate*.

*Proof*.

$$e_{\text{COV}}\left(t\right)={\dot{\theta }}_{\text{ref}}-\dot{\theta }\left(t\right)={\dot{\theta }}_{\text{ref}}-\frac{1}{\Delta t}\left[\theta \left(t\right)-\theta \left(t-\Delta t\right)\right]$$ (40)

$$=\frac{1}{\Delta t}\left({\dot{\theta }}_{\text{ref}}\;\Delta t-\theta \left(t\right)+\theta \left(t-\Delta t\right)\right)$$ (41)

$$=\frac{1}{\Delta t}e_{\text{AP}}\left(t\right).$$ (42)

**Lemma 4.3.3.** *OAC is equivalent to AP and COV, if AP and COV employ an adaptive tangent reference velocity* ${\dot{\theta }}_{\text{ref}}≜\dot{\theta }\left(t-\Delta t\right)$.

*Proof*.

$$e_{\text{OAC}}\left(t\right)=-\ddot{\theta }\left(t\right)=\frac{1}{\Delta t}\left(\dot{\theta }\left(t\right)-\dot{\theta }\left(t-\Delta t\right)\right)$$ (43)

$$=-\frac{1}{\Delta t^{2}}\left(-\theta \left(t\right)+\theta \left(t-\Delta t\right)+\dot{\theta }\left(t-\Delta t\right)\Delta t\right)$$ (44)

By setting ${\dot{\theta }}_{\text{ref}}:=\dot{\theta }\left(t-\Delta t\right)$ and re-arranging we obtain

$$e_{\text{OAC}}\left(t\right)=\frac{1}{\Delta t^{2}}\left({\dot{\theta }}_{\text{ref}}\;\Delta t-\theta \left(t\right)+\theta \left(t-\Delta t\right)\right)$$ (45)

$$=\frac{1}{\Delta t^{2}}e_{\text{AP}}.$$ (46)

Theorem 4.3.1 then follows from Lemma 4.3.2 and Lemma 4.3.3.

We see that *e*_OAC_ and *e*_AP_ both assume a fixed ${\dot{\theta }}_{\text{ref}}$ and only differ by a constant scaling factor—the square of the frame rate. In contrast, OAC updates the tangent reference velocity ${\dot{\theta }}_{\text{ref}}$ in every time step using the tangent velocity from the previous time step. We hypothesize that OAC will generate more complex behavior since the implicitly optimized reference velocity will drift with the current observation (since velocity and acceleration constraints will prevent the agent for optimally zeroing the control error). Moreover, OAC will be more affected by sensing noise as it needs to numerically compute $\ddot{\theta }$, the second derivative of tan *α*.

Based on the insights from the previous sections, we now present novel implementations of Chapman’s strategy. These implementations use a bang-bang controller to zero the control error *e*, but define *e* in such a way to address shortcomings of previously suggested implementations, COV/AP and OAC.

#### 4.3.4 Constant optical velocity with Initial Observation (COV-IO)

The first implementation exploits Lemma 4.2.5 which state that the tangent reference ${\dot{\theta }}_{\text{ref}}$ can be estimated robustly when observing $\dot{\theta }$ at the beginning of the ball trajectory. This overcomes the issue of COV and AP of assuming a fixed a value for ${\dot{\theta }}_{\text{ref}}$. We term this implementation *COV-IO*, for *Constant Optical Velocity with Initial Observation of ${\dot{\theta }}_{\text{ref}}$*.

We further increase the robustness of COV-IO by filtering both the estimate ${\dot{\theta }}_{\text{ref}}$ as well as the current reference velocity $\dot{\theta }$ (detailed in the supplementary material S2 Text).

#### 4.3.5 Combining COV and OAC—COV-OAC

The second implementation exploits Lemma 4.3.3, which establishes a relation between COV and OAC. It allows us leverage the fact that OAC does not require explicit computation of ${\dot{\theta }}_{\text{ref}}$ and combine it the COV’s advantage of not having to compute a second derivative of the tangent velocity $\ddot{\theta }$. The reason we want to avoid the second derivative is because it amplifies random noise, in particular at high control rates.

We thus suggest *COV-OAC*, which estimates ${\dot{\theta }}_{\text{ref}}$ using the delayed tangent velocity $\dot{\theta }\left(t-\Delta_{{\dot{\theta }}_{\text{ref}}}\right)$:

$$\begin{array}{c} e_{\text{COV}-\text{OAC}}\left(t\right)=\dot{\theta }\left(t\right)-\dot{\theta }\left(t-\Delta_{{\dot{\theta }}_{\text{ref}}}\right). \end{array}$$ (47)

If $\Delta_{{\dot{\theta }}_{\text{ref}}}=\Delta t$, the controller reduces to a standard (unnormalized) OAC controller, otherwise if $\Delta_{{\dot{\theta }}_{\text{ref}}}=t$ we obtain COV-IO. In practice, we choose a value $0<\Delta_{{\dot{\theta }}_{\text{ref}}}⪡t$ that approximates OAC close enough while still compensating high-frequent random fluctuations. To increase robustness to noise even further, we use moving average filters for estimating $\dot{\theta }\left(t\right)$ and $\dot{\theta }\left(t-\Delta_{\dot{\theta }}\right)$, similar to COV-IO.

We hypothesize that COV-IO and COV-OAC result in the most robust implementations of Chapman’s strategy, in particular when random noise is present. To validate this hypothesis, we will compare our two novel implementations of Chapman’s strategy to COV(/AP), OAC and the Cartesian controllers in simulated experiments in Section 4.4.

#### 4.3.6 Remark on the biological plausibility of Chapman’s strategy

We would like to remark that the existence of COV-IO and COV-OAC counters an argument brought forward by [50] regarding the biological plausibility of Chapman’s strategy. The authors conclude that humans do not use Chapman’s strategy because they lack the ability to visually perceive accelerations accurately enough for control. The fact that COV-IO and COV-OAC only rely on the computation of (averaged) velocities clearly disproves this argument.

#### 4.3.7 Angular control in three dimensions: Constant bearing angle

So far, we have been concerned with the two-dimensional scenario. As explained in Section 2.7.1, the bearing angle can be used to extend any implementation of Chapman’s strategy to three dimensions. The idea is to combine the control output of Chapman’s strategy, which we denote by *u*_Chapman_ with a second control output *u*_*CBA*_ which maintains a constant bearing angle *β* between the agent and the ball. The two control outputs are then applied to perpendicular axes, formally

$$\begin{array}{c} u=u_{\text{Chapman}}v+u_{\text{CBA}}v_{⊥}, \end{array}$$ (48)

where **v** = [*v*_*x*_, *v*_*z*_]^*T*^ = [*a*_*x*_ − *b*_*x*_, *a*_*z*_ − *b*_*z*_]^*T*^ is the vector pointing from the agent to the ball’s shadow on the ground plane, **v**_⊥_ = [*v*_*z*_, −*v*_*x*_] is the orthogonal vector on the ground plane (see Fig 1A) and

$$\begin{array}{c} u_{\text{CBA}}=k_{\text{CBA}}\;\dot{\beta }. \end{array}$$ (49)

for some constant gain *k*_*CBA*_.

**Implementation**: We apply two techniques to make CBA robust to random noise. First, we compute $\dot{\beta }$ by finite differencing at some *low* frequency $\frac{1}{\Delta_{\beta }},\Delta_{\beta }<\Delta t$:

$$\begin{array}{c} \dot{\beta }\left(t\right)=\frac{\beta \left(t\right)-\beta \left(t-\Delta_{\beta }\right)}{\Delta_{\beta }}. \end{array}$$ (50)

Second, we apply a moving-average filter similar to OAC-COV (Section 4.3.5). In total, this introduces three hyperparameters, the time delay Δ_*β*_ as well as the averaging window sizes *h*_*β*_ and $h_{\beta_{\text{ref}}}$.

#### 4.3.8 Chapman and the Markov property

We conclude the theoretical analysis of the angular control strategies by discussing their relationship to the Markov property. As discussed before, the Markov property is an important prerequisite for using optimal control and reinforcement learning. For example, the theoretical guarantees for LQG do not hold if we cannot provide a Markov state representation. Therefore, it is interesting to study whether the angular representation used by Chapman’s strategy (depending on the implementation, *θ*, $\dot{\theta }$ or $\ddot{\theta }$) fulfills the Markov property. If this was the case, we might consider applying LQG or a similar variant to the angular representation.

However, it turns out that neither *θ*, $\dot{\theta }$ nor $\ddot{\theta }$ are Markov. The full proofs are lengthy and can be found in the supplementary material (S1 Text). As an example, we provide an intuitive explanation for why *θ* is not Markov. The idea is to show that, even if the agent does not move and the environment is deterministic, we cannot predict the future *θ*(*t*′) from a single observation *θ*(*t*) for any *t*′ > *t*. Such a case can easily be found: we just look at different initial conditions *ν*_0_ ≠ *ν*_1_, *φ*_0_ ≠ *φ*_1_ that result in trajectories *θ*_1_(*t*), *θ*_2_(*t*) which cross at some point *θ*_1_(*t*_1_) = *θ*_2_(*t*_2_) but have different slopes ${\dot{\theta }}_{1}\left(t_{1}\right)\neq {\dot{\theta }}_{2}\left(t_{2}\right)$. Similar type of reasoning can be carried out to show that $\dot{\theta }$ and $\ddot{\theta }$ are not Markov, either.

We will discuss the implications of this finding in Section 7.

### 4.4 Experiments

The goal of the following section is to verify our theoretical findings about the Cartesian and angular controllers for ball catching. To that end, we conduct a large set of experiments testing both types of controllers in simulated experiments under a variety of different perturbations. These experiments will show that both types of controllers result in optimal catching performance in the ideal case but that there is “no free lunch” for any of the two types: whereas Cartesian controllers are impeded by systematic modeling errors, resulting from wrong assumptions about drag, the performance of angular controllers degrades when high amounts of Gaussian noise is present.

#### 4.4.1 Experimental set-up

We now present the experimental set-up, including a description of the physical simulation environment, the set of tested perturbations and the controller implementations.

All code has been made publicly available at https://github.com/shoefer/ball_catching.

**Simulation**: In our simulation we choose the ball parameters to standard baseball rules with mass *m*_*b*_ = 0.15 kg and radius *r* = 0.0366 m. When simulating drag, we assume experimentally established parameters for baseballs [51]: air density $\rho =1.293\frac{\;\text{kg}}{\;m^{3}}$, frontal area *A* = *πr*^2^ and drag coefficient *c*_*w*_ = 0.5. To model the agent, we use the approximate values of olympic sprinters who can run with a maximal acceleration of roughly ${\ddot{a}}_{\text{max}}=3\frac{m}{s^{2}}$ and reach a maximum velocity of ${\dot{a}}_{\text{max}}=11\frac{m}{s}$ [52, 53]. The simulation runs at a frame rate of 60 Hz (time constant $\Delta t=\frac{1}{60}\;s$).

**Initial Conditions**: For each strategy and noise scenario we test different ball-agent configurations. In all configurations, the agent is in principle able to catch the ball, given its velocity and acceleration constraints, but we include extreme cases where the agent must operate at its limits to be successful.

In *two dimensions*, we use the configurations suggested by [43]. The initial ball launching angle is always set to $\varphi =\frac{\pi }{4}$, and we vary (i) the initial ball velocity *ν* = {20, 24, 28, 32, 36, 40} and (ii) the distance of the agent to the ball’s impact point *D*_0_ ∈ {−15, −10, −5, 0, 5, 10, 15} (*D*_0_ = *a*_0_ − *R*, see Fig 1B).

In *three dimensions*, we additionally vary the initial position of agent in the direction orthogonal to the ball trajectory (*z*-axis in Fig 1B), parametrized by the angle *ψ*. We report the averaged results over $\psi \in \{\frac{\pi }{16},\frac{\pi }{8},\frac{\pi }{4},\frac{\pi }{2}\}$.

In the cases where random noise is applied to the ball trajectory, we must ensure that the initial conditions regarding the agent’s position hold. We therefore run the simulation twice in order to determine the actual ball impact point: we first record the ball trajectory including the actual noise, and then position the agent at the right distance to the actual impact point and replay the ball trajectory recorded in the first run.

**Measuring Performance Criteria**: In order to evaluate the performance of a controller we use the terminal distance cost $L_{\text{terminal}\;\text{distance}}$ defined in Section 2.3.

**Perturbations** We distinguish two types of perturbations.

**Perturbations of the** ***ball trajectory***: These include (i) drag, (ii) wind gust (wind over short period of time) and (iii) turbulence (spin, Magnus force, and so on).

**Perturbations of the** ***agent’s sensory and motor capabilities***: These include (iv) sensor noise, (v) motor noise, (vi) sensorimotor delay and (vii) reduced control rate. Table 1 summarizes all perturbations and their implementations.

**Table 1:** Overview of implemented perturbations.

Perturbation	Implementation	Values
**Ball**		
*Drag (air resistance)*	See Eq (2).	(i) off, (ii) on.
*Wind gust*	Force **F**_wind_ applied for 0.1 s after 0.4*T* (trajectory duration).	(i) *headwind* **F**_wind_ = (−8, 2, 0)^*T*^ (*opposite to* flight direction, lifting), (ii) *tailwind* **F**_wind_ = (8, 2, 0)^*T*^ (*in* flight direction, lifting).
*Turbulence*	Gaussian noise (***μ*** = **0**,**Σ** = *σ* **I**) applied to ball position.	(i) *σ* = 0.001, (ii) *σ* = 0.01, (iii) *σ* = 0.1
**Agent**		
*Sensor noise*	Gaussian noise (***μ*** = **0**,**Σ** = *σ*λ_*d*_||**a** − **b**||^2^ **I**) applied to *sensed* ball position/velocity, modulated with agent-ball distance.	(i) *σ* = 0.001, (ii) *σ* = 0.01, (iii) *σ* = 0.1, λ_*d*_ = 0.05.
*Motor noise*	Gaussian noise (***μ*** = **0**,**Σ** = *σ* **I**) added to **u**.	(i) *σ* = 0.001, (ii) *σ* = 0.01, (iii) *σ* = 0.1.
*Sensorimotor delay*	Time delay for agent to receive visual stimulus.	(i) 0 ms (off) (ii) 200 ms (average reaction time of humans at college age, [54]), (iii) 400 ms (extreme case, as in [16]).
*Control rate*	Simulation time step Δ*t*.	(i) 60 Hz, (ii) 10 Hz.

To keep the number of experimental conditions at reasonable size, we vary the perturbations to (iii) turbulence, (iv) sensor noise and (v) motor noise jointly, that is always use the same standard deviation *σ* for them. We therefore refer to these three perturbations as *Gaussian perturbations*.

The main set of experiments studies the influence of *individual perturbations* (listed in Table 1). Additionally, we test a *worst case* scenario with Gaussian noise *σ* = 0.1, wind and 400 ms delay (we run this scenario both with and without drag and reduced control rate).

In order to obtain statistically significant results, we run every controller five times for every experiment.

#### 4.4.2 Controller implementations

We test the *angular controllers* COV, OAC, COV-IO and COV-OAC and the *Cartesian controllers* (i)LQG (Section 4.1) and MPC [16]. For the controllers that have external parameters, we conducted preliminary experiments and determined the set of parameters that performed good both at ideal and perturbed cases. Additionally, we make the model parameters used to generate the Cartesian controllers explicit by writing (i)LQG_no drag_ for a model that assumes an ideal ball trajectory, (i)LQG_drag:baseball_ for a model that assumes drag parameters of a baseball, and so on.

Furthermore, we need to take special care for evaluating the MPC controller. First, it uses a slightly more complex agent representation. To guarantee a fair comparison to the other methods, we tested a variety of different parameters and selected the ones that yielded the best average performance (detailed in the supplementary material S2 Text). Second, the implementation provided by the authors is computationally expensive. To be able to collect sufficient amounts experimental data, we only simulate the strategy at a frame rate of 10 Hz (as in the original paper). We therefore mark the strategy with a * in all comparisons where it is evaluated with a different frame rate than the other strategies.

Table 2 summarizes the controllers and the parameters used.

**Table 2:** Controller implementations. For angular controllers only parameters for the two-dimensional case are shown, see text for parameters in three dimensions.

Name	Description	Parameter Settings
**Angular Controllers**		
**OAC**	Optical acceleration cancellation	-
**COV**	Constant optical velocity (equivalent to AP, see Lemma 4.3.2)	${\dot{\theta }}_{\text{ref}}=0.2$
**COV-IO**	COV with initial observation of ${\dot{\theta }}_{\text{ref}}$	$h_{\dot{\theta }}=\frac{1}{12}\text{s}$, $h_{{\dot{\theta }}_{\text{ref}}}=\frac{5}{12}\text{s}$.
**COV-OAC**	COV with delayed ${\dot{\theta }}_{\text{ref}}$ estimation	$h_{\dot{\theta }}=\frac{1}{12}\text{s}$, $h_{{\dot{\theta }}_{\text{ref}}}=\frac{5}{12}\text{s}$, $\Delta_{{\dot{\theta }}_{\text{ref}}}=\frac{1}{6}\text{s}$.
**Cartesian Controllers**		
**(i)LQG**^nodrag^, **(i)LQG**^drag:baseball^, **(i)LQG**^drag:soccer^	(Iterative) linear-quadratic Gaussian control with different drag dynamics	Cost function terms: *w*_terminal distance_ = 1000, *w*_control effort_ = 0.1. Full dynamics and parameters for (extended) Kalman filter are given in the supplementary material (S2 Text).
**MPC**^nodrag^	Model-predictive control in belief space [16]	*F*_1_ = 7.5, *F*_2_ = 7.5, *M* = 10^−3^, *Ω* = 10^−15^. The internal model is equivalent to the one used by (i)LQG *without drag*.

**Controller Implementation in 3D**: The Cartesian controllers (i)LQG and MPC can be readily applied to both the two- and the three-dimensional case. To extend the angular strategies, we combine them with constant bearing angle (CBA, Section 4.3), using parameters Δ_*β*_ = 0.15 s and $h_{\beta }=\frac{1}{6}\;s$.

#### 4.4.3 Results

We now present the main results of our experiments. We study the results as to whether they confirm our two main hypotheses: that both angular and Cartesian controllers are optimal in the ideal case, but that different perturbations affect them in different ways.

Since the results for the two- and three-dimensional cases are largely equivalent, most of the plots depicted in the following only show results from the two-dimensional case. For the reader’s convenience, the results in three dimensions are provided in the supplementary material (S1 Fig).

**Fig 8:**
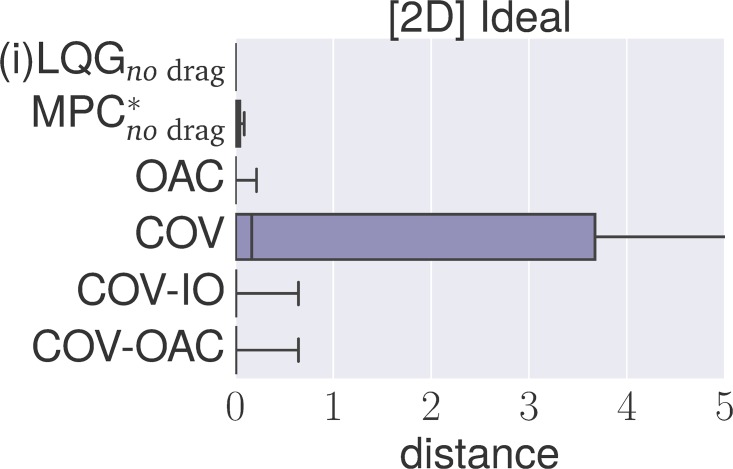
Comparison of ball catching strategies in 2D for ideal case.

**Fig 9:**
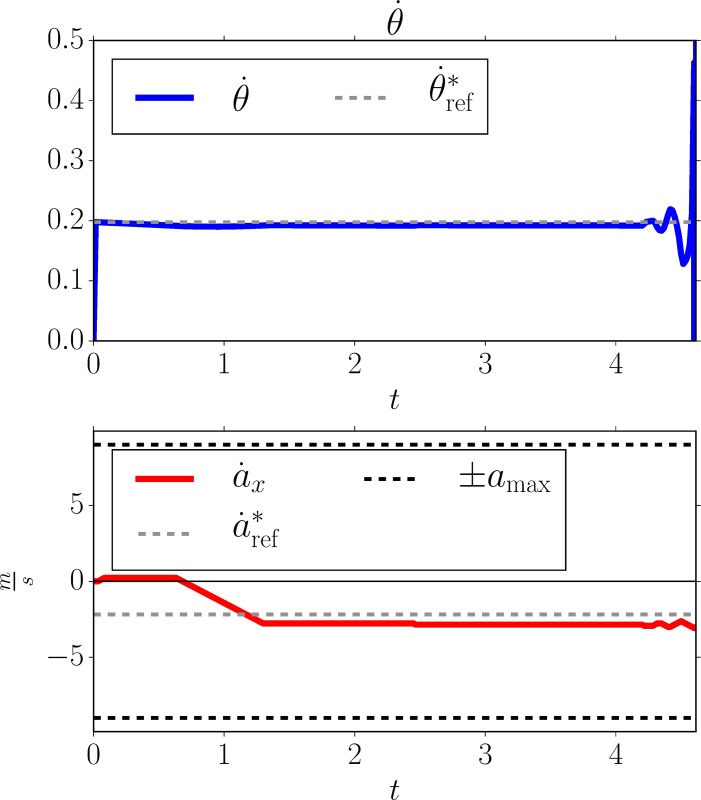
COV-IO: Agent velocity and tangent of vertical viewing angle velocity, initial conditions $D_{0}=10m,\nu =32\frac{m}{s},\varphi =\frac{\pi }{4}$.

**Both Angular and Cartesian Control Solve the Ideal Case**: We first study the performance of the controller implementations in the ideal case, without perturbations. Fig 8 shows the terminal distance averaged over all initial conditions. We see that (i)LQG, MPC, OAC, COV-IO and COV-OAC perform optimally, achieving close to zero terminal distance. This result confirms our theoretical analysis: (i)LQG and MPC guarantee optimal performance since their assumptions are fulfilled. COV-OAC is optimal as it is a direct implementation of Chapman’s strategy (Section 4.3). The good performance of COV-IO, which sets ${\dot{\theta }}_{\text{ref}}$ according to the initial observation, has been predicted by Lemma 4.2.5. We also see that for COV-IO the agent attains a value close to the consistent agent velocity ${\dot{a}}_{\text{ref}}$ (Fig 9), as proved in Lemma 4.2.3. Only COV performs inferior because it uses a fixed value for ${\dot{\theta }}_{\text{ref}}$—as predicted by Corollary 4.2.4. We therefore exclude COV from the following experiments.

We conclude that all sensible implementations of the angular and Cartesian controllers succeed in the ideal scenario.

**Fig 10:**
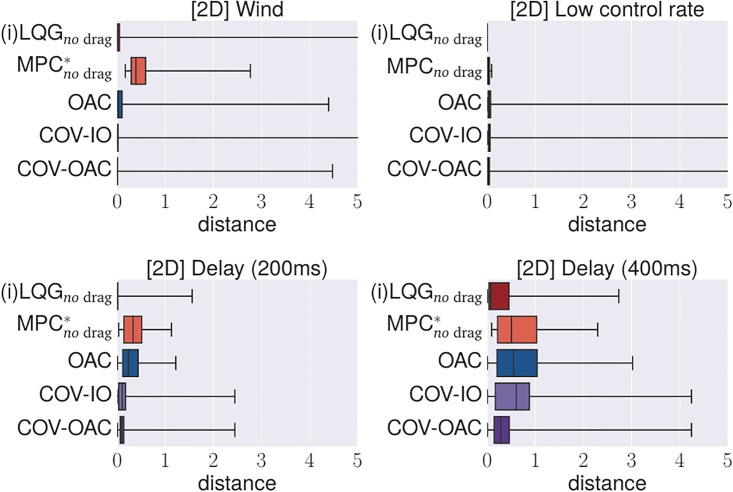
Comparison of ball catching strategies in 2D: Sensitivity to different *individual perturbations*.

**Fig 11:**
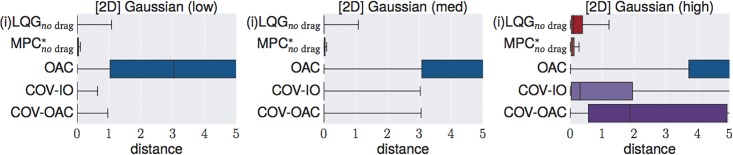
Comparison of ball catching strategies in 2D: Sensitivity to *Gaussian perturbations*.

**No Free Lunch for Angular and Cartesian Control**: Next, we inspect the influence of perturbations on controller performance. Figs 10 and 11 depict the average performance in the two-dimensional case when applying perturbations individually.

First, we observe that most of the angular and Cartesian controllers are largely unaffected by a reduced control rate, wind, and sensorimotor delays (Fig 10). Only delays of 400 ms pose a problem for the controllers, but solely because the delayed observation in combination with the agent’s motion constraints render extreme initial configurations unsolvable. We will thus focus on Gaussian perturbations (turbulence, sensor and motor noise) and drag in the following.

**Angular controllers are impaired by high Gaussian perturbations**: Fig 11 shows that OAC performs significantly worse for low amounts of Gaussian noise, whereas COV-IO and COV-OAC perform optimally for the low and medium setting. The reason is that COV-IO and COV-OAC operate on the first derivative of *θ* and use moving average filtering, whereas OAC operates on the second derivative, which amplifies noise much stronger. However, the performance of COV-IO and COV-OAC degrades for high Gaussian noise, whereas (i)LQG only degrades a bit and MPC is barely affected.

**Fig 12:**
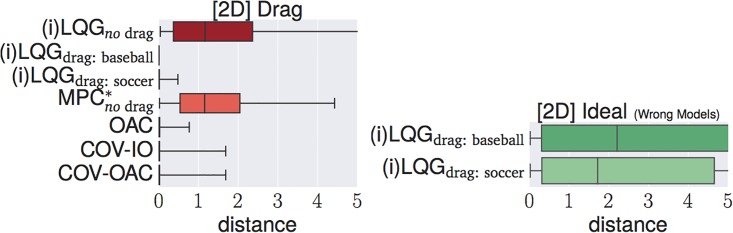
Comparison of ball catching strategies in 2D for *drag*, and performance of (i)LQG with wrong drag model in ideal case.

**Fig 13:**
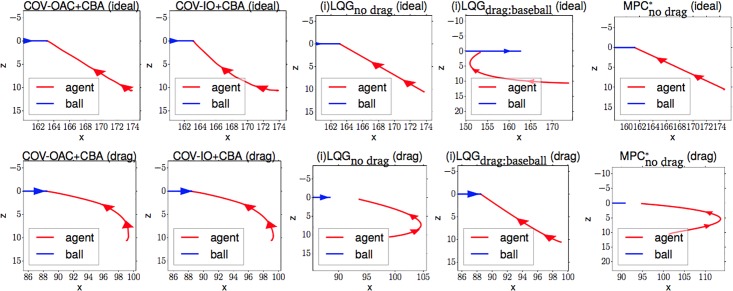
Agent trajectories (bird’s eye view) for different strategies in the three-dimensional case with and without drag. Initial conditions are set to $D_{0}=15m,\nu =40\frac{m}{s},\varphi =\frac{\pi }{4},\psi =\frac{\pi }{4}$. Top row: *with drag*. Bottom row: *without drag*.

**Cartesian controllers are impaired by wrong model assumptions regarding drag**: The plots in Fig 12 show that the performance of all Cartesian controllers degrades significantly when the underlying model assumes wrong drag parameters. Fig 13 illustrates the behavior of the controllers with wrong drag assumptions in three dimensions. If the Cartesian controller assumes drag but we do not simulate it, the controller systematically overestimates the range *R* of the trajectory (Fig 13, top row). In the opposite case, the Cartesian controllers underestimate the range (Fig 13, bottom row).

**Fig 14:**
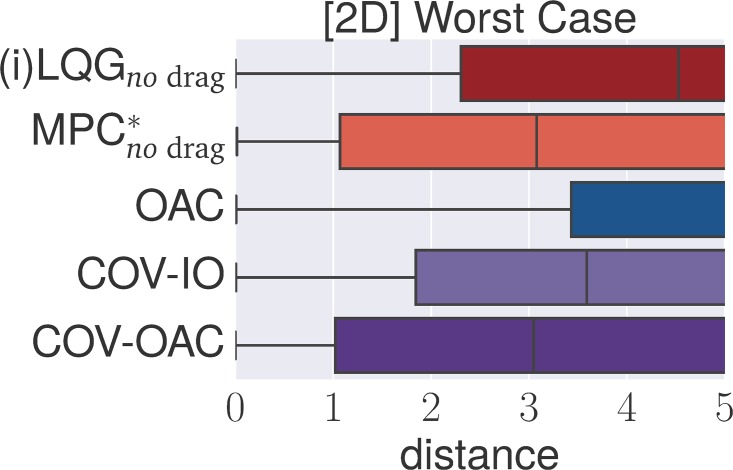
Sensitivity to *combinations of perturbations* (2D).

**Fig 15:**
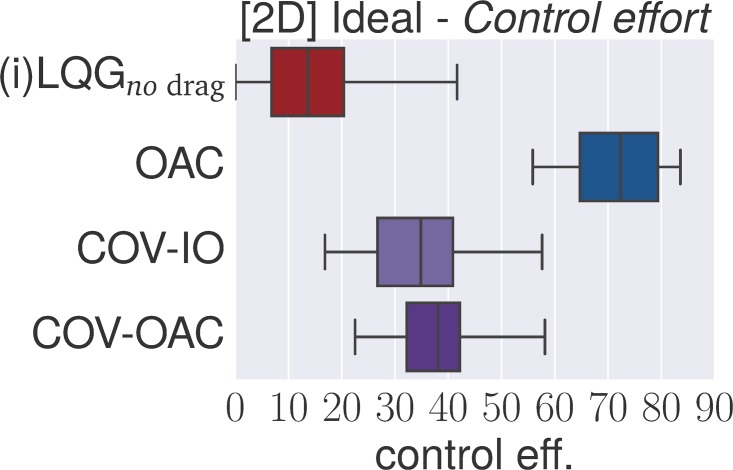
Control effort in *ideal case* (2D).

**Fig 16:**
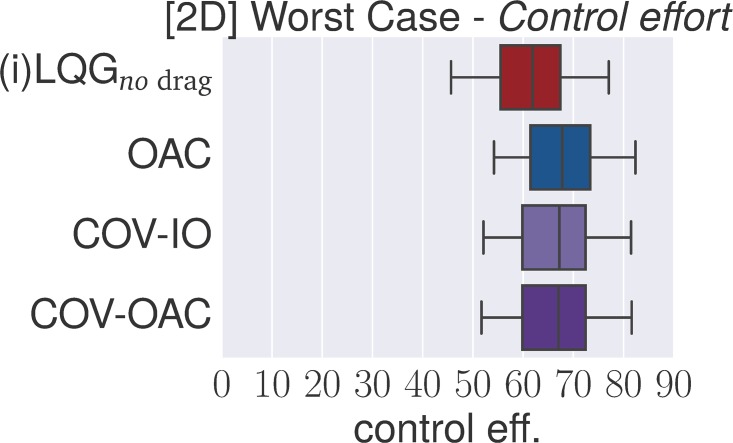
Control effort affected by *combinations of perturbations* (2D).

Finally, Fig 14 shows how the controllers are affected by combinations of perturbations. We see that in the worst case scenario where all perturbations are applied with the highest value, both types of controllers perform similarly bad. Figs 15 and 16 show that the same holds true for the control effort. This confirms our result that there is no free lunch for any type of controller: angular controllers are impaired by high Gaussian noise, Cartesian controllers by wrong drag parameters, yet both perform equally bad when averaged over the various scenarios.

## 5 On the optimality of Chapman’s angular control strategy

The previous section provided new insights about the differences between generalist and specialist approaches to ball catching: we showed that angular controllers generalize better to systematic and Cartesian controllers better to Gaussian perturbations of the ball trajectory. This raises the question about the main cause for these differences.

The goal of this section is to show that optimality is not a criterion: therefore, optimization is a valid tool to obtain both generalist *and* specialist, “heuristic” solutions for decision making problems. We have discussed in Section 3 that this result is known for the non-sequential decision making setting, and our result extends it to the sequential decision making setting.

To make this point, we show that the specialist approach to ball catching, the angular control strategy, is optimal in its “preferred” environment: an environment with systematic, but without Gaussian perturbations. We show this by conducting the following experiment: the agent has to *learn* how to catch the ball (i) in an environment without Gaussian perturbations, but the agent is only given access to (ii) observations from a (simulated) camera and to (iii) a reward signal proportional to its catching performance. This setting leaves it open to the agent how to use the observation to solve the ball catching task. Hence, if we can show that the agent learns a successful controller *and* that the learned controller structurally resembles any of the angular control strategies presented in the previous section, we show that the angular control strategy is optimal for this environment.

### 5.1 Learning setting

We now provide a high-level overview of the learning setting and the structure of the argument that we show the optimality of the angular control strategy. An in-depth treatment of all technical details will be given in Section 5.2.

In the following, we confine ourselves to the two-dimensional ball catching scenario (Section 4.4.1). The reason is that the previous section provided strong theoretical guarantees for both Cartesian and angular control in this case, and it results in a lower-dimensional camera image, facilitating the analysis of what is learned.

We will begin our argument by providing an explicit list of biases used in the learning experiments. The purpose of this list is to enable the reader to convince herself that all employed biases and assumptions are task-general and not tailored to the ball catching problem—and thus do not implicitly rely on any heuristics.

Next, we will explain the camera model that simulates observations, and show how to use reinforcement learning to learn a controller that is directly applied to the observations. We will then show how to manually implement the angular controllers COV-IO and COV-OAC as baselines. Importantly, we will use the same camera model and controller parametrization as for the learned controllers. This will allow us to compare the parameters of the learned controllers with the baselines, and thus show that an equivalence between these holds. This equivalence will show that the reinforcement learning procedure is able to learn an angular controller that forms an instance of the specialist, heuristic ball catching approach.

#### 5.1.1 Biases for learning

In our experiment, we rely on a set of assumptions and learning biases which we summarize in Table 3. The reason why this list might seem long at first sight is because most of these biases are commonly left implicit in applications of optimization and machine learning. For example, linear functions are very common as they can be learned efficiently and are effective at solving many tasks while still being easy interpret. Their interpretability enables us to show that the agent indeed learns an angular controller.

**Table 3:** Biases used in the reinforcement learning experiment for ball catching.

Bias	Description	Where Explained
Simulated camera	To provide the input to the agent we simulate a camera that generates a 1D gray-scale image.	[13], Section 5.1.2, Fig 17
Full observability	In order to observe the ball at any position, we assume that the agent has a 180 degree field of view.	Section 5.1.2
No high-frequency Gaussian noise	The agent does not have to deal with any form of high-frequency Gaussian noise, but only drag.	–
Augmented input	We augment the camera image given as input by applying a *time-embedding*, including images of previous time-steps, and temporal pixel-wise image derivatives. We test various combinations of input augmentations, considering them as features to be selected by the learner.	[55], Section 5.1.2
Linear P-control policy	The agent maps pixels to motor outputs (accelerations) using a *linear* mapping. The mapping is factored into two components: a pixel-specific weight vector and a scalar proportional gain factor.	Section 5.1.3
Curriculum learning	To guide optimization we start training with simple initial conditions and proceed to more complex ones as soon as the agent’s learning performance progresses.	[56], Section 5.1.3
Vision-related regularization	We incorporate prior knowledge about the camera model by regularizing the policy, enforcing similar weights for adjacent pixels.	[57], Section 5.1.3

Inevitably, these assumptions impose a bias on the policy and thus exclude certain types of solutions. In particular, the assumption of a linear policy implies that it is stationary, which excludes any type of Cartesian controller (Section 4.1.4). Although we might be able to learn a Cartesian controller by removing assumptions (for example using a recurrent neural network) it is *not* the scope of this experiment to show that reinforcement learning is more likely to find an angular or Cartesian controller. Instead, we aim to show that we can state an optimization problem with reasonable, task-general assumptions, and that the solution to this problem is a heuristic angular controller. This shows that angular controllers can be considered as optimal with respect to these assumptions.

We turn to an overview of the learning experiment. For every component of our scenario, we will refer back to the list of biases presented in Table 3 and detail how each bias is implemented. To guide the reader, we will mark each reference to a bias with an underscore.

#### 5.1.2 Camera model

**Fig 17:**
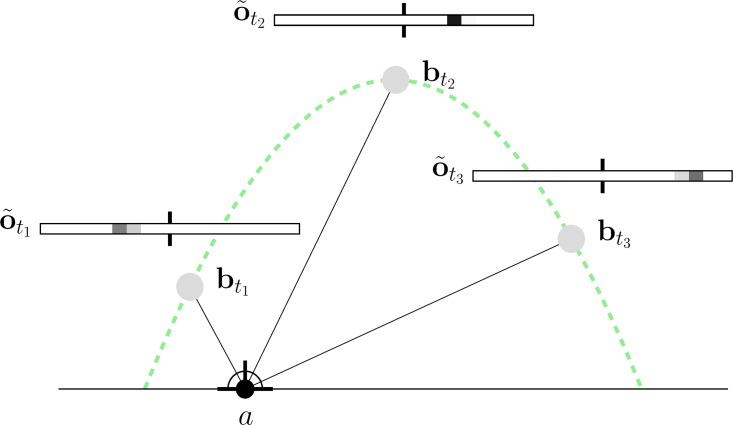
Exemplary raw observations generated by the simulated camera model. The agent *a* observes the ball *b*_*t*_ at different times *t* and receives a 1D image with a field of view of 180°.

In order to perform learning directly on sensor data we equip the agent with a simulated camera sensor, illustrated in Fig 17. The camera provides a full 180°view, with the ball occupying a certain set of pixels in the camera image depending on its position in the environment. We term this image the *raw observation* and denote it by $\tilde{o}\in R^{N}$ with dimensionality *N*. The 180° view ensures that the full observability bias is fulfilled. This bias is helpful in two regards: it is based on the same position-controlled agent model used in the previous section and thus allows us to transfer our previous insights for analyzing the learned policies. Further, fully observable problems are much easier to solve using learning than partially observable problems.

All implementational details about the camera model are provided in the supplementary material (S3 Text).

**Input Augmentation**: The successful controllers presented in the previous section operate both on an input signal as well as on higher derivatives of this signal—for example, the angular controllers operate on *θ*, $\dot{\theta }$ or $\ddot{\theta }$, and the Cartesian controllers on $a,b,\dot{a}$ and $\dot{b}$. To enable the learner to come up with a successful control policy, too, we *augment* the raw observation such that the policy is able to compute derivatives, if necessary. The controller is thus provided with these *augmented observations* as input. In order to distinguish them from the raw observations $\tilde{o}$, we denote the augmented observations by **o**.

We augment the observation in two ways. First, we combine the current image $\tilde{o}\left(t\right)$ with a temporal derivative of the image $\dot{\tilde{o}}\left(t\right)$, computed using image differencing $\dot{\tilde{o}}\left(t\right)=\tilde{o}\left(t\right)-\tilde{o}\left(t-1\right)$. The notation for referring to this type of augmentation is $o=\tilde{o}⊕\dot{\tilde{o}}$, where ⊕ denotes the concatenation of two vectors. Second, we add a second delayed camera image to the observation: either the image at initial time *t*_init_ or the image that is delayed by *t*_delay_.

Enriching the input in this way implements the augmented input bias. Note that we also evaluate the performance operating directly on the non-augmented observations. The complete list of tested augmentations is provided in Table 4.

**Table 4:** Observation augmentations.

	Time-embedding		
Observation type	*Current* (*t*)	*Current & initial* (*t*, *t*_init_)	*Current & delayed* (*t*, *t* − *t*_delay_)
*Observation* $\tilde{o}$	$o\left(t\right)=\tilde{o}\left(t\right)$	$o\left(t\right)=\tilde{o}\left(t\right)⊕\tilde{o}\left(t_{\text{init}}\right)$	$o\left(t\right)=\tilde{o}\left(t\right)⊕\tilde{o}\left(t-t_{\text{delay}}\right)$
*Derivative* $\dot{\tilde{o}}$	$o\left(t\right)=\dot{\tilde{o}}\left(t\right)$	$o\left(t\right)=\dot{\tilde{o}}\left(t\right)⊕\dot{\tilde{o}}\left(t_{\text{init}}\right)$	$o\left(t\right)=\dot{\tilde{o}}\left(t\right)⊕\dot{\tilde{o}}\left(t-t_{\text{delay}}\right)$
*Obs. & Derivative* $\tilde{o},\dot{\tilde{o}}$	$o\left(t\right)=\tilde{o}\left(t\right)⊕\dot{\tilde{o}}\left(t\right)$	$\begin{array}{ll} o\left(t\right) & =\tilde{o}\left(t\right)⊕\dot{\tilde{o}}\left(t\right)\\ & ⊕\tilde{o}\left(t_{\text{init}}\right)⊕\dot{\tilde{o}}\left(t_{\text{init}}\right) \end{array}$	$\begin{array}{l} o\left(t\right)=\tilde{o}\left(t\right)⊕\dot{\tilde{o}}\left(t\right)\\ ⊕\tilde{o}\left(t-t_{\text{delay}}\right)⊕\dot{\tilde{o}}\left(t-t_{\text{delay}}\right) \end{array}$

#### 5.1.3 Reinforcement learning on observations

The task of the agent is to learn a controller *π*(**o**) = *u*, called *policy* in the reinforcement learning setting, which maps every observation **o** to a control signal *u*. (Here the control signal *u* is scalar since we only consider the two-dimensional scenario.) We now explain how we define the policy *π* and use reinforcement learning to find *π*.

**Linear Policy**: In our experiments, we exploit the linear P-control policy bias by decomposing *π* such that it mimics the structure of a P-controller. This allows the learner to compute the control input and the control gain separately:

$$\begin{array}{c} \pi \left(o\right)=vw^{T}o, \end{array}$$ (51)

where v∈R denotes the scalar *gain factor* and $w\in R^{N}$ the *pixel-specific weight vector*; *N* corresponds to the size of the (augmented) observation **o**.

We train one policy per augmented observation type and evaluate which one perform best (according to their terminal distance at impact time).

**Policy Search Using CMA-ES**: We apply model-free, policy-based reinforcement learning, using the stochastic optimization method *Covariance Matrix Adaption—Evolutionary Strategy (CMA-ES)* [58, 59]. Evolutionary methods such as CMA-ES optimize the policy *π* by maintaining a set of candidate policies, and by iteratively sampling new policies based on their performance with respect to a given cost $L_{\text{ES}}$ until converging to a local optimum of this cost.

To apply CMA-ES to the ball catching problem we could, in principle, set $L_{\text{ES}}$ to the terminal distance cost $L_{\text{terminal}\;\text{distance}}$, which we already used in Chapter 4 to assess the performance of angular and Cartesian controllers. However, applying this cost directly results in slow or bad performance, as explained in the following. Therefore, we use the *regularized worst-case terminal distance* cost

$$L_{\text{ES}}=L_{\text{terminal}\;\text{distance}}^{†}+\lambda L_{\text{spatial}},$$ (52)

which consists of a modified version of the terminal distance cost $L_{\text{terminal}\;\text{distance}}^{†}$ and an additional regularization term $L_{\text{spatial}}$, weighted by hyperparameter *λ*. These two terms will be explained in the following. They address practical learning issues of applying CMA-ES to the ball catching problem and incorporate the biases curriculum learning and vision-related regularization.

**Optimizing for Multiple Initial Conditions**: Our ultimate goal is to find a policy *π* that works well for a wide range of initial conditions. However, $L_{\text{terminal}\;\text{distance}}$ only evaluates the performance of *π* for a *single* initial condition. We therefore evaluate every candidate policy considered by CMA-ES on *a set of initial conditions* I, and compute the *worst-case terminal distance cost* for this set:

$$\begin{array}{c} L_{\text{terminal}\;\text{distance}}^{†}=\max_{i\in I}L_{\text{terminal}\;\text{distance}}. \end{array}$$ (53)

By considering only the initial condition where the policy performs worst, we avoid that the optimization procedure settles on a local optimum where the policy performs very well on some, but very bad on other initial conditions.

**Curriculum Learning with CMA-ES**: Optimizing for the worst-case cost $L_{\text{terminal}\;\text{distance}}^{†}$ is costly and thus increases the runtime of CMA-ES if the set of initial conditions I is very large. To resolve this problem, we use curriculum learning to adapt the set of initial conditions I in every iteration of CMA-ES: we sort all initial conditions by difficulty in ascending order. We then define sets $I_{0},I_{1},\ldots ,I_{M}$ of increasing size as follows: $I_{0}=\left\{i_{0}\right\}$ only contains the simplest initial condition and we add more difficult conditions one-by-one to every subsequent set $I_{1},\ldots ,I_{M}$. To use these sets, we apply CMA-ES on $I_{i}$, starting with *i* = 0, and proceed to $I_{i+1}$ once the cost $L_{\text{terminal}\;\text{distance}}^{†}$ drops below a pre-defined threshold (in our experiments $L_{\text{terminal}\;\text{distance}}^{†}<0.5$).

**Vision-Related Regularized Training Cost**: Although $L_{\text{terminal}\;\text{distance}}^{†}$ is better-suited for learning than $L_{\text{terminal}\;\text{distance}}$ it still results in poor performance because we do not exploit any knowledge about the fact that we learn a policy that is applied to a camera image **o**. An important property of camera sensors is the spatial arrangement of pixels: it results in any visible object being projected on a set of *adjacent* rather than disconnected pixels in **o**. Exploiting this knowledge as a vision-related regularization bias for learning is very common in computer vision [57], and we do so too by defining the *spatial continuity regularization* term

$$\begin{array}{c} L_{\text{spatial}}=\frac{1}{N-2}\sum_{i=0}^{N-2}{\left(w_{i+1}-w_{i}-\frac{1}{N-2}\sum_{j=0}^{N-2}\left(w_{j+1}-w_{j}\right)\right)}^{2}, \end{array}$$ (54)

where {0, …, *N* − 1} denote the indices of the image **o** and *w*_*i*_ denotes the policy’s weight associated with the *i*-th image pixel. We can write this formula more concisely if we define *Var* as the (uncorrected) sample variance and **w**′ as the *spatial* derivative over vector **w**:

$$\begin{array}{c} L_{\text{spatial}}=\text{Var}\left[w^{\prime }\right]. \end{array}$$ (55)

#### 5.1.4 Angular baseline policies

To analyze whether the previously outlined reinforcement learner indeed finds an angular, “heuristic” controller, we will compare the weights of the learned policies to the weights of two *baseline policies*.

The baseline policies mimic the COV-IO and COV-OAC controllers (Section 4.3), and use the same camera model and the same two-step linear controller as the learned policies. This makes it easy to directly compare the policies by inspecting their weights *v* and **w**.

We now illustrate how to compute the baseline policies, using the example of COV-IO. We know from Section 4.3 that COV-IO is implemented by zeroing the control error $e_{\text{COV}-\text{IO}}=\dot{\theta }\left(t_{\text{init}}\right)-\dot{\theta }\left(t\right)$ using a bang-bang controller. Therefore, the COV-IO baseline policy must be able to compute the quantities $\dot{\theta }\left(t_{\text{init}}\right)$ and $\dot{\theta }\left(t\right)$ from the augmented observation and subtract them from each other. Therefore, we compute a linear mapping $\Omega \left(o\right)=w_{\Omega }^{T}o$ such that $\Omega \left(o\right)\propto \dot{\theta }\left(t_{\text{init}}\right)-\dot{\theta }\left(t\right)$, for some suitable augmented observation **o**. The COV-IO control output can then be computed by applying bang-bang control on Ω(**o**).

We show in the supplementary material (S3 Text) how to choose **o** and how to compute Ω to implement COV-IO and COV-OAC directly on observations. Importantly, we will compare the mapping Ω (in particular the pixel-specific parameter vector **w**_Ω_) with the learned policy parameters to decide whether they resemble the COV-IO and COV-OAC strategies.

To avoid confusion with the controllers implemented in the previous section, operating directly on *θ*, $\dot{\theta }$ and higher derivatives, we refer to the two baseline policies operating on observations as COV-IO^**o**^ and COV-OAC^**o**^.

### 5.2 Experimental setup

We now describe the technical details of our learning experiment.

#### 5.2.1 Simulation and scenario

**Simulation**: We run the dynamic simulation with a time constant of $\Delta t=\frac{1}{60}\;s$, using the same simulation as in the previous experiments described in Section 4.4.1.

**Camera Sensor**: For the camera model, we use resolution *ρ* = 0.27 which results in a size of *N* = 18 pixels for the raw observation $\tilde{o}$. We test different input augmentations by varying the *type of observation* (observation only, derivative only or both) and by adding a second delayed camera image to the observation, which we call *time-embedding* (no delay, observation at *t*_init_ or at *t* − *t*_delay_). We test different variants, such as adding the image at initial time *t*_init_ or the image that is delayed by *t*_delay_. We use the following parameters: $t_{\text{initial}}=\frac{1}{20}\;s$, and $t_{\text{delay}}=\frac{5}{6}\;s$.

Note that depending on type of input augmentation, the dimensionality of **o** varies between *N* = 18, *N* = 36 and *N* = 64.

This results in the nine different augmentations summarized in Table 4, which we all test in our experiments.

**Performance and Testing Procedure**: We test the success of each strategy in the same way as in the experiments presented in Chapter 4: using the terminal-distance cost $L_{\text{terminal}\;\text{distance}}$ evaluated on the full set of initial conditions.

**Initial Conditions and Perturbations**: To train the agent we choose the initial conditions to be a *reduced set of the ones* used in the previous experiments (see Section 4.4.1). Reducing the set has two purposes: first, it accelerates learning, second, it tests whether the learner overfits or is able to generalize to a range of unseen conditions.

The reduced set of initial conditions I uses the same lauching angle $\varphi =\frac{\pi }{4}$ as the full set, but only varies the initial ball velocity by *ν* = {20, 30, 40} $\frac{m}{s}$ and the initial distance of the agent to the ball’s impact point *D*_0_ ∈ {−15, −7.5, 0, 7.5, 15} *m*. Additionally, we vary whether drag forces apply to the ball or not. In total, this results in 30 unique combinations of initial conditions.

#### 5.2.2 Reinforcement learning

We now briefly detail the settings of the reinforcement learner.

**CMA-ES Hyperparameters**: For CMA-ES, we use a candidate parameter set of size 10, and set the maximum number of CMA-ES iterations to 10000. We set the initial variance for *v* to $\sigma_{v}^{2}=15\times 10^{10}$ and for **w** to $\sigma_{w}^{2}=15$. Note that setting the value of $\sigma_{v}^{2}$ very high allows the agent to learn either a proportional or an approximation of a bang-bang controller.

As convergence criteria, we allow a tolerance of 0.1 with respect to variance in the cost $L_{\text{terminal}\;\text{distance}}^{†}$ and of 0.1 in the input parameters. Moreover, we vary the spatial regularization parameter *λ* ∈ {0.01, 0.1}.

Note that we also tested adding L1- and L2-regularization to $L_{\text{ES}}$ but this did not result in improved performance.

**Curriculum**: To construct the curriculum of initial conditions we sort the set of (reduced) initial conditions according to initial distance *D*_0_, initial ball velocity *ν* and the presence of drag (no/yes), resulting in a sequence of 30 sets of initial conditions of increasing difficulty.

Every time CMA-ES converges for one set of initial conditions from the curriculum, we proceed to the next set and multiply the variance parameters *σ*_*v*_ and *σ*_**w**_ by 1.2. In preliminary experiments this gave the better performance as it avoided premature convergence, that is convergence before the end of the curriculum is reached.

We also compare to optimization without a curriculum, that is setting I to all initial conditions; however, this consistently leads to significantly worse performance and we thus omit the analysis of these results in the following section.

For each combination of input augmentation, regularization parameter setting and initial condition selection (curriculum or not), we run CMA-ES five times with different random seeds and report the best result for every run.

### 5.3 Results

**Fig 18:**
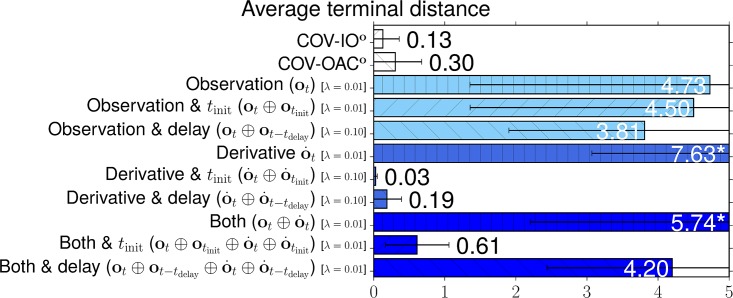
Average terminal distance when learning policies on different types of observations. Values annotated by a star indicate that the corresponding bar exceeds the bounds of the figure.

Fig 18 shows the performance of the linear policies learned by CMA-ES, applied to the nine different types of augmented observations. It also includes the performance of the baselines COV-IO^**o**^ and COV-OAC^**o**^. We see that the type of observation augmentation has the biggest influence on the result. Moreover, we see that the best results are achieved when using a spatial regularization *λ* = 0.1.

We now study which type of observation augmentations perform best. We see that the best results are achieved when using $o={\dot{\tilde{o}}}_{t}⊕{\dot{\tilde{o}}}_{t_{\text{init}}}$, resulting in a terminal distance of 0.03, followed by $o={\dot{\tilde{o}}}_{t}⊕{\dot{\tilde{o}}}_{t-t_{\text{delay}}}$ with terminal distance of 0.19. None of the other observation augmentation types yields competitive results, except for the policy that uses $o={\tilde{o}}_{t}⊕{\tilde{o}}_{t_{\text{init}}}⊕{\dot{\tilde{o}}}_{t}⊕{\dot{\tilde{o}}}_{t_{\text{init}}}$, achieving 0.61 average terminal distance. We hypothesize that it performs slightly worse than the two best policies because the augmented observation is more high-dimensional, containing both $\tilde{o}$ and $\dot{\tilde{o}}$. This leads to overfitting and thus decreases performance.

Interestingly, the two best policies also slightly outperform the supervised baselines. They seem to have adapted better to the imperfections of the sensor with respect to the control task, rather than only adapting to the task of predicting the supervised signal.

#### 5.3.1 Learned policies are equivalent to COV-IO and COV-OAC

The quantitative analysis shows that it is possible to learn a successful ball catching policy directly on observations, using generic reinforcement learning. We now turn to a qualitative analysis, by studying *what* type of policy has been learned, and whether one of the policies implements one of the angular controllers presented in the previous section. To answer this question, we will study the control outputs and the parameters learned by the two best policies and compare them to the parameters of the baselines policies.

The two best policies resulted from applying CMA-ES on the augmented observations $o={\dot{\tilde{o}}}_{t}⊕{\dot{\tilde{o}}}_{t_{\text{init}}}$ and $o={\dot{\tilde{o}}}_{t}⊕{\dot{\tilde{o}}}_{t-t_{\text{delay}}}$, we thus focus on these ones. In the following, we state our main results and provide all details in the supplementary material (S3 Text).

First, we observe that the CMA-ES policies compute a bang-bang control policy: for both the value of *v* is very high (|*v*|>10^11^). This facilitates our comparison because we implemented the baseline policies as bang-bang controllers, too. Next, we compare the policies with respect to the control output they compute. We use the data used to train the baseline policies, discretize the output of both controllers *y* ∈ {−1, 0, 1} and use a zero-one loss

$$\begin{array}{c} L\left(\Omega ,{\left\{\left(o^{\left(i\right)},y^{\left(i\right)}\right)\right\}}_{i=1,\ldots ,N}\right)=\frac{1}{N}\sum_{i=0}^{N}\left(1-1\{\Omega \left(o^{\left(i\right)}\right)=y^{\left(i\right)}\}\right), \end{array}$$ (56)

to compare the outputs (1{⋅} denotes the indicator function which evaluates to 1 if the expression inside the brackets is true and to 0 otherwise). We obtain a similarity of 0.89 for COV-IO^**o**^ vs. $o={\dot{\tilde{o}}}_{t}⊕{\dot{\tilde{o}}}_{t_{\text{init}}}$ and 0.77 for COV-OAC^**o**^ vs. $o={\dot{\tilde{o}}}_{t}⊕{\dot{\tilde{o}}}_{t-t_{\text{delay}}}$. This shows the outputs are similar, in particular for COV-IO^**o**^, although not exactly the same.

**Fig 19:**
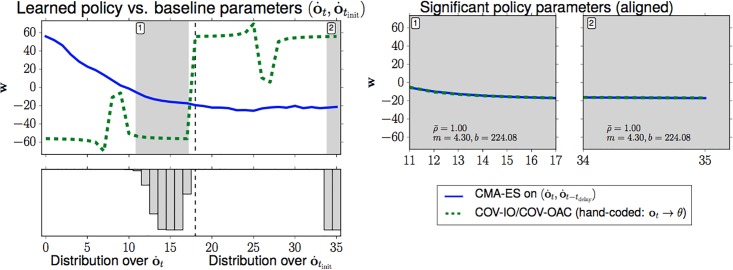
Analysis of policy parameters, with CMA-ES applied to $o_{t}=\left({\dot{o}}_{t},{\dot{o}}_{t_{\text{init}}}\right)$. $\bar{\rho }$ denotes the correlation coefficient, *m* and *b* the slope and intercept of the linear fit.

**Fig 20:**
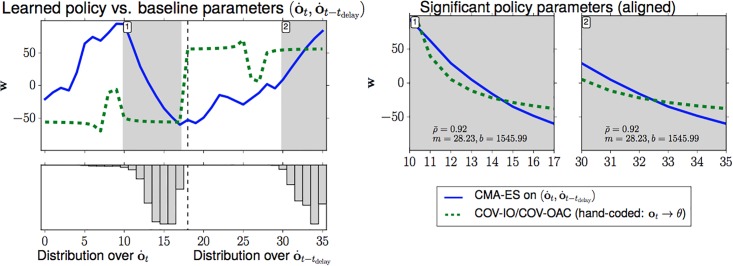
Analysis of policy parameters, with CMA-ES applied to $o_{t}=\left({\dot{o}}_{t},{\dot{o}}_{t-t_{\text{delay}}}\right)$.

Finally, we compare the pixel-specific weight vectors **w**. Figs 19 and 20 show a comparison of the learned policy weights (blue solid curves) to the weights of the baseline policies (green dashed curves). At first sight, they do not look similar at all. We hypothesize that this results from the fact that not all entries in **w** are relevant for control. This can be the case if the ball always occupies the same area in the camera image, for example because it never passes above the agent’s head. In this case, the ball always appears in the right part of $\tilde{o}$ and $\dot{\tilde{o}}$.

To investigate this hypothesis, we apply the learned policies on the set of all initial conditions and compute a histogram over each pixel in **o**. This histogram is shown in the left bottom halves of Figs 19 and 20. It confirms our hypothesis that the ball never passes the agent’s head because the left parts are completely empty. This is a result of the fact that the ball is always thrown from the same side and that the agent moves in such a way that it never *lets* the ball pass its head. This is an indication for the stable catching behavior—but also for some form of “overfitting” to the learning scenario.

Since the ball always occupies a certain part of the image, all weights outside this part of the image are irrelevant for performance. We thus compare the learned weights to the baseline weights only with respect to the relevant part of the image. The result is shown in Figs 19 and 20, upper right half. We see that, after accounting for the offset induced by focusing on a limited set of weights, the weights align almost perfectly for COV-IO^**o**^, and approximately for COV-OAC^**o**^. This means that, CMA-ES applied to $o={\dot{\tilde{o}}}_{t}⊕{\dot{\tilde{o}}}_{t_{\text{init}}}$ has learned a controller that is highly similar to COV-IO^**o**^, and a controller similar to COV-OAC^**o**^ when applied to $o={\dot{\tilde{o}}}_{t}⊕{\dot{\tilde{o}}}_{t-t_{\text{delay}}}$.

#### 5.3.2 Conclusion

Our analysis shows that CMA-ES learns policies that are highly similar to the COV-IO and COV-OAC angular controllers presented in Section 4.3. The learned policies merely differ in the fact that they do not generalize to the setting when the ball is thrown from the left. However, we see no principle reason why the learned policies should not generalize to this slightly more complex version if we train on more initial conditions. We thus conclude that model-free reinforcement learning is able to find angular control policies. This implies that the *angular control strategy is an optimal solution to the two-dimensional ball catching problem without high-frequency Gaussian perturbations*.

## 6 Discussion

Our results show that the generalist and specialist approaches yield two very different solutions to the ball catching problem, each optimal and more appropriate for a different environmental condition. Our in-depth theoretical and empirical analysis has identified a variety of properties along which the two solutions differ, which we summarize in Table 5.

**Table 5:** Revised comparison of differences between generalist and specialist solutions to ball catching.

	*Generalist*	*Specialist*
**How to find a solution?**	(Model-based) optimal control	Model-free reinforcement learning
**Input representation**	*Cartesian:* Agent/ball position and velocity, **a**, $\dot{a},b,\dot{b}$	*Angular:* Tangent of vertical viewing angle *θ*, $\dot{\theta },\ddot{\theta }$, bearing angle *β*
**Input = Markov state?**	yes	no
**Controller type**	PD-control	Bang-bang / P-control
**Stationary controller?**	No	Yes
**Optimal wrt. to environmental condition**	High-frequency Gaussian noise	Low-frequency systematic noise (drag)

We now discuss implications and possible extensions of our work. We structure the discussion along the four main contributions of our study, stated in the introduction: (i) the implications of no approach being superior in solving the ball catching problem under all environmental conditions, (ii) the advantages and disadvantages of model-based and model-free control, (iii) the possibility of generalizing our optimality results to different ball catching scenarios, and (iv) the role of representations for ball catching and decision making problems. In every section, we derive hypotheses how we might find a solution to ball catching that generalizes across all relevant experimental conditions.

### 6.1 No free lunch for ball catching

The main result of our work is that angular controllers are robust to systematic deviations of the ball’s trajectory, such as drag, whereas the Cartesian controllers cope better with high-frequency Gaussian noise. We can relate this, at least in an informal way, to the no free lunch theorem [60]: every strategy makes implicit assumptions about the problem, but this come at the price of degraded performance on other problem variants.

This raises the question about whether one type of perturbation, drag or high-frequency Gaussian noise, is more relevant in realistic settings. The answer to this question is not obvious and can ultimately only be given by real-world experiments. However, conducting controlled experiments in a baseball setting [12] is difficult, and no robots for ball catching beyond small distance throws [33] have been developed yet.

### 6.2 Model-based vs. Model-free control

We saw that the Cartesian controllers suffer from a well-known disadvantage of model-based control in general: the sensitivity to modeling errors [9]. However, the Cartesian approach leverages the model to make predictions when observations are noisy, using Bayesian filtering. This type of filtering reduces Gaussian noise more effectively than the temporal averaging employed by the model-free angular controllers, and thus copes better with high amounts of Gaussian noise.

Assuming that both types of perturbations are relevant, the question arises how to extend or combine model-free angular and model-based Cartesian control to cope with both types of perturbations:

**Cartesian control with hidden variable estimation**: One option to decrease the sensitivity to drag of the Cartesian controllers is by treating the drag coefficient as a hidden random variable and performing inference over it [61]. However, such an approach might come at the cost of high computational complexity.

**Model-based angular control**: Another option is to make the angular controller robust to high-frequency noise by developing a model-based variant of angular control. Although this is impeded by the angular representation not being Markov, practically feasible solutions might exist: when taking into account multiple instead of only a single derivative of the vertical viewing angle, the resulting representation might violate the Markov assumption only moderately. Then, an approximate forward model could be stated and combined with an optimal control method like LQG or MPC.

We will discuss another option based on a different representation in Section 6.4.

### 6.3 Optimal ball catching in complex settings

Our analysis has shown that the two solutions are not only superior, but even optimal under the different environmental conditions. We now discuss how these optimality guarantees could be transferred to more complex variants of the ball catching problem.

**Chapman’s strategy in three dimensions**: Our theoretical analysis of the angular controllers provided insight about the two-dimensional scenario but it left open how to extend our theoretical results to the three-dimensional case, for example when using Chapman’s strategy with CBA (Section 4.3.7). Although our empirical results clearly indicate that OAC generalizes to the three-dimensional case when paired with CBA, an analytical proof to support our empirical results is required.

Similarly, the reinforcement learning experiments in Chapter 5 only solved the two-dimensional ball catching problem. Addressing the three-dimensional scenario necessitates a way of coping with the increased dimensionality of the visual sensory input. This requires substituting the linear policy used in our experiments with a more expressive function class such as (convolutional) neural networks. These networks have become a de-facto standard in processing and learning from visual inputs [24] and reinforcement learning [2].

**Partially observable ball catching variants**: An interesting question is how the controllers generalize to partially observable variants of the ball catching problem. One such variant is presented by [16]: it requires the agent to turn away from the ball and run open-loop in order to catch the ball. [16] suggested a method to solve this problem, but, as shown in Section 4.4.1, it is based on the Cartesian representation and thus fails for systematic, non-Gaussian perturbations of the ball trajectory such as drag. This result raises the question whether solutions based on the angular representation exist for the more complex ball catching problem, too. Although similar control problems can be addressed with local reactive behavior [62] and a (model-free) extensions of the angular controller presented here, incorporating a field of view exists [8], each of these approaches requires the ball to remain in the field of view for the entire time, preventing them from being applied to the partial observability case. Only if a model-based version of the angular controller (see previous section) exists it might be able to deal with the partial observability scenario.

### 6.4 The role of representations for ball catching and decision making

Our work shows that whether we choose the Cartesian or the angular representation has a significant impact on the complexity and the performance of the resulting controller. Therefore, we propose to treat representations as a first-class citizen for solving any type of decision making problems. In such a *representation-centric view*, the representation “dictates” all relevant choices, such as the necessity of a model, the control type, etc., and thus influences computational complexity and success under different environmental conditions. The representation leaves out unnecessary information and exposes the relevant aspects of the problem space—in the case of ball catching, it pretty much *is* the solution.

Therefore, a representation-centric view also offers another option for finding a solution that is robust to all realistic perturbations: a controller optimal under both high-frequency noise and drag might require a representation different from the ones studied here—be it with model-free or model-based control.

How could we find such a representation? We suggest to leverage recent advances in representation learning [63] and deep reinforcement learning to study this question. Representation learning methods are *explicit* methods in the sense that they optimize a learning objective that characterizes useful properties of representations. How to define “usefulness” in a generalizable way is an open research question, and promising candidates for ball catching might be methods that exploit *physical prior knowledge* about how agents move in the real world [64] as well as methods that explicitly optimize representations to be useful for for model-based control [65, 66]. In contrast, deep reinforcement learning methods are *implicit* in the sense that they directly optimize the control objective (catching success in our case) and build intermediate representations in the neural network that is trained to map from inputs to outputs. We are curious to see how these methods would perform in one of the generalized ball catching settings and what kind of representations they would learn.

## 7 Conclusion

In this work, we studied the ball catching problem with the goal of investigating the relationship between generalist and specialist approaches to decision making. We found out that neither of the two approaches is superior and that each approach can be considered optimal under a different environmental conditions. We showed that the key difference between these approaches has to be sought in the representation, angular vs. Cartesian, which has the most significant impact on the agent’s ability to solving the ball catching problem.

We conclude this paper by arguing that finding the right solution to a decision making or control problem is orthogonal to the generalist and specialist approach, and thus requires a reconciliation of these views: (i) We need generalist, optimality-based learning to solve problems that we cannot solve directly through engineering. But since brute-force, uninformed learning requires large amounts of data even for problems as simple as the ball catching problem (ii) we must also embrace the specialist view. It is required to gain insights into specific problems, which we can first turn into task-specific biases and eventually into biases that generalize over entire sets of problems. Our view that solving decision making and control problems requires a trade-off between biases and learning is clearly supported by the bias-variance and the no free lunch theorems in supervised learning, and we believe that future work should—rather than arguing in favor of one or the other extreme—study how to effectively balance biases and learning in decision making and control.

## Supporting information

S1 Fig Comparison of Cartesian and angular control: Results from 3D experiments. Comparison of ball catching strategies in 3D; sensitivity to individual perturbations and a combination of perturbations.
(PDF)

S1 Text Proofs: Angular representations violate Markov property. Formal proofs of the result stated in Section 4.3.8.
(PDF)

S2 Text Comparison of Cartesian and angular control: Supplementary material for experiments. Formal explanation of the moving averaging computation and parameters for LQG, iLQG and MPC control strategies used in Section 4.4.
(PDF)

S3 Text On the optimality of Chapman’s strategy: Supplementary material for experiments. Detailed explanation of camera simulation used in Section 5, as well as how the baseline policies in this section are computed and compared to the learned policies.
(PDF)

S4 Text Introduction to Linear-quadratic Gaussian control. A concise and formal introduction to Linear-Quadratic Gaussian control (LQG), including linear-quadratic regulators, Kalman filters and its nonlinear extensions iterative LQG (iLQG) and extended Kalman filters, respectively.
(PDF)
