# Supplementary material for: No free lunch in ball catching: A comparison of Cartesian and angular representations for control
Source: PLoS One. 2018 Jun 14;13(6):e0197803. doi: 10.1371/journal.pone.0197803 (PMC6002113; doi:10.1371/journal.pone.0197803)
Supplement: S1 Fig — Comparison of ball catching strategies in 3D; sensitivity to individual perturbations and a combination of perturbations. (PDF) [file pone.0197803.s001.pdf]

No Free Lunch in Ball Catching: A Comparison of Cartesian and Angular Representations for Control
Supplementary Material (S1 Fig)
Comparison of Cartesian and Angular Control: Results from 3D Experiments

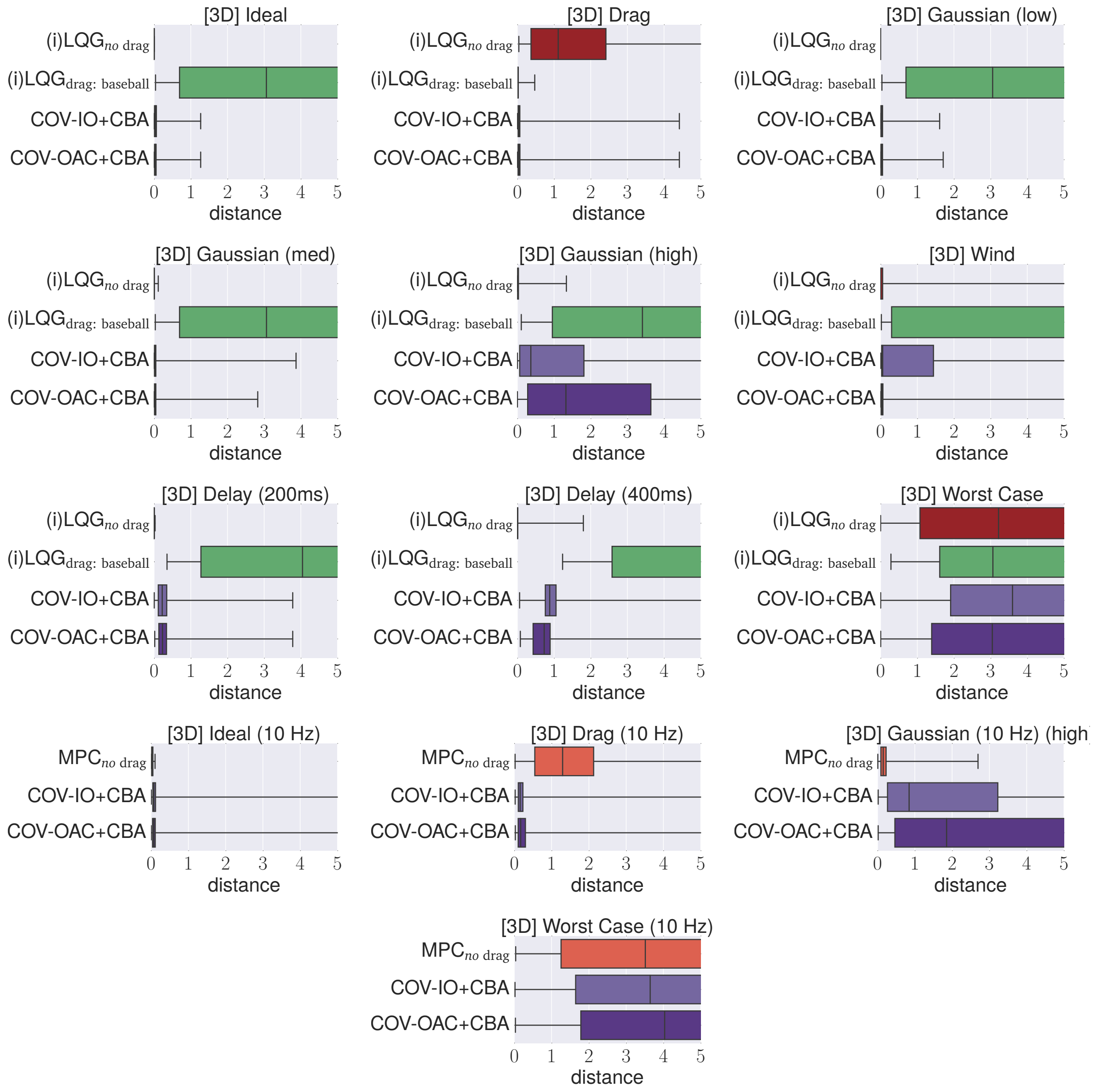

Comparison of ball catching strategies in 3D: sensitivity to individual perturbations and a combination of perturbations.
