# Supplementary material for: No free lunch in ball catching: A comparison of Cartesian and angular representations for control
Source: PLoS One. 2018 Jun 14;13(6):e0197803. doi: 10.1371/journal.pone.0197803 (PMC6002113; doi:10.1371/journal.pone.0197803)
Supplement: S1 Text — Formal proofs of the result stated in Section 4.3.8. (PDF) [file pone.0197803.s002.pdf]

No Free Lunch in Ball Catching: A Comparison of Cartesian and Angular Representations for Control
Supplementary Material (S1 Text)
Proofs: Angular Representations Violate Markov Property

Contents

1 Introduction **1**

2 Non-Markov θ **2**

3 Non-Markov Derivative of θ **3**

4 Non-Markov Second Derivative of θ **3**

1 Introduction

In the following, we will prove that the representations used by angular controllers for ball catching are not Markov. To that end, we will prove the following theorem:

Theorem 1.1 (Angular representations are not Markov)**.** *Given $x \in \{\theta, \dot{\theta}, \ddot{\theta}\}$ with dynamics $x_A(t)$ governed by initial conditions $A = \{\nu_A, \phi_A\}$ and agent trajectory $a(t)$, there exist initial conditions $B = \{\nu_B, \phi_B\}$, $A \neq B$, time steps t_A, t_B, and an agent trajectory $a(t)$ such that two following conditions are fulfilled:*

(i) $x_A(t_A) = x_B(t_B)$,

(ii) $\dot{x}_A(t_A) \neq \dot{x}_B(t_B)$.

Our ansatz to proof Theorem 1.1 is by finding counterexamples showing that the angular representations $\{\theta, \dot{\theta}, \ddot{\theta}\}$ violate the Markov property.

We know a quantity $x(t)$ fulfills the Markov property if for $t > t_n > \ldots > t_1$:

$$P(x(t) \,|\, x(t_n)) = P(x(t) \,|\, x(t_n), \ldots, x(t_1)). \tag{1}$$

Intuitively, this means that it is sufficient to rely solely on $x(t_n)$ for predicting any future state $x(t)$; adding more information about the past of $x(t)$ does not make the prediction better. If x is deterministic, Eq. (1) is equivalent to the statement that there exists exactly one $x(t)$ for which

$P(x(t) \,|\, x(t_n)) = 1$. In this case, it is sufficient to show that the latter condition does not hold, for example by finding at least two cases where the same $x(t_n)$ has different derivatives $\dot{x}(t_n)$.

Therefore, our ansatz is as follows. In the ball catching scenario, the dynamics of $\theta, \dot{\theta}$ and $\ddot{\theta}$ are governed by the initial conditions $\{\nu, \phi\}$, corresponding to the initial ball velocity and angle, and by the agent's motion $a(t)$. For the sake of the analysis, we assume deterministic dynamics. Without loss of generality, we will further assume a trivial agent trajectory, with fixed D and a static agent, $a(t) = R + D = a_0$. It is sufficient to analyze this special case, since for a representation to be Markov means that it must be Markov for all possible agent trajectories. This facilitates our analysis as the dynamics of $x \in \{\theta, \dot{\theta}, \ddot{\theta}\}$ then only depend on the initial conditions. We make explicit that the dynamics of x are governed by initial conditions A by writing $x_A(t)$. Theorem 1.1 then stataes that we can find different sets of initial conditions A and B, $A \neq B$, and time steps t_A, t_B, such that (i) the values of x at the different initial conditions and time steps are identical, $x_A(t_A) = x_B(t_B)$, but such that (ii) their first derivatives differ $\dot{x}_A(t_A) \neq \dot{x}_B(t_B)$.

Note that we are only considering one-dimensional angular representations, not combinations of derivatives of θ. The reason is that the heuristic control strategies considered here only rely on one-dimensional representations.

All proofs have been verified using the symbolic solver SymPy [1], and the code has been made publicly available as a jupyter notebook in the following git repository:
`https://github.com/shoefer/ball_catching`.

2 Non-Markov θ

We begin with the simplest case, θ, and prove the following lemma:

Lemma 2.1 (θ is not Markov)**.** *For $\theta_A(t)$ governed by initial conditions $A = \{\nu_A, \phi_A\}$ and agent trajectory $a(t)$, there exist initial conditions $B = \{\nu_B, \phi_B\}$, $A \neq B$, time steps t_A, t_B, and an agent trajectory $a(t)$ such that (i) $\theta_A(t_A) = \theta_B(t_B)$ but (ii) $\dot{\theta}_A(t_A) \neq \dot{\theta}_B(t_B)$.*

Proof. We know that θ is linear if the agent is waiting at the landing point ($D = 0$), and that the trajectory of θ has slope $\dot{\theta}$. Therefore, we consider this special case, and search for two different sets of initial parameters $A = \{\nu_A, \phi_A\}$, $B = \{\nu_B, \phi_B\}$ and time steps t_A and t_B such that (i) $\theta_A(t_A) = \theta_B(t_B)$ and (ii) $\dot{\theta}_A(t_A) \neq \dot{\theta}_B(t_B)$.

We start by computing the values of θ and its derivatives for the special case $D = 0$:

$$\theta = \frac{gt}{2\nu \cos(\phi)}, \tag{2}$$

$$\dot{\theta} = \frac{g}{2\nu \cos(\phi)}. \tag{3}$$

The equations show that the slope $\dot{\theta}$ is constant and solely depends on the initial parameters (and gravity). Therefore, the only way to make them different, and thus fulfill condition (ii), is to choose different initial parameters. Without loss of generality, we assume $\nu_A \neq \nu_B$ and $\phi_A = \phi_B$. We now need to find time steps t_A and t_B such that equation (i) is fulfilled, $\theta_A(t_A) = \theta_B(t_B)$. By solving this equation for t_A, we obtain:

$$t_A = \frac{\nu_A t_B}{\nu_B} \tag{4}$$

Therefore, (i) $\theta_A(t_A) = \theta_A(\nu_A {\nu_B}^{-1} t_B) = \theta_B(t_B)$, but by construction (ii) $\dot{\theta}_A(t_A) \neq \dot{\theta}_B(t_B)$. This shows that θ is not Markov. □

3 Non-Markov Derivative of θ

Next, we show that $\dot{\theta}$ is not Markov either:

Lemma 3.1 ($\dot{\theta}$ is not Markov)**.** *For $\dot{\theta}_A(t)$ governed by initial conditions $A = \{\nu_A, \phi_A\}$ and agent trajectory $a(t)$, there exist initial conditions $B = \{\nu_B, \phi_B\}$, $A \neq B$, time steps t_A, t_B, and an agent trajectory $a(t)$ such that (i) $\dot{\theta}_A(t_A) = \dot{\theta}_B(t_B)$ but (ii) $\ddot{\theta}_A(t_A) \neq \ddot{\theta}_B(t_B)$.*

Proof. This case is a bit more involved as we cannot assume that the agent waits at landing point, that is we cannot assume $a(t) = R$, $D = 0$. The reason is that for this case $\dot{\theta}$ is constant and thus $\ddot{\theta}$ is zero, which makes the representation Markov in a trivial way.

Our ansatz is to create an explicit dependency between ν_A and ν_B as well as t_A and t_B, and then solve for the parameters such that they fulfill (i) and (ii). Without loss of generality, we will keep $\phi_A = \phi_B$, and define $\nu_B = \nu_A + \varepsilon$ by some $\varepsilon \neq 0$, and define $t_B = t_A + \delta$ for some $\delta \neq 0$ and solve for δ.

Additionally, we need to ensure that $D \neq 0$ (and $D \neq -R$; it must not coincide with the ball starting position because then θ is linear, too). Since we are only interested in a counterexample, we can set D to any suitable value and thus use $D = -\frac{R}{2}$. This means the agent is standing exactly in the middle between the ball's starting and landing point.

By applying all of the aforementioned constraints, we can compute $\theta(t)$ and its first and second derivatives:

$$\theta(t) = \frac{gt\left(\nu_A \sin(\phi) - \frac{1}{2}gt\right)}{\nu\left(\nu\sin(\phi) - gt\right)\cos(\phi)}, \tag{5}$$

$$\dot{\theta}(t) = \frac{g\left(gt\left(\nu\sin(\phi) - \frac{1}{2}gt\right) + \left(-\nu\sin(\phi) + gt\right)^2\right)}{\nu\left(-\nu\sin(\phi) + gt\right)^2\cos(\phi)}, \tag{6}$$

$$\ddot{\theta}(t) = \frac{\nu g^2 \sin^2(\phi)}{\left(\nu\sin(\phi) - gt\right)^3\cos(\phi)}. \tag{7}$$

By solving $\ddot{\theta}_A(t_A) = \ddot{\theta}_B(t_B)$ for δ, we obtain two conjugate solutions, from which we choose the positive one. As δ results in s complex formula, we omit writing them it explicitly here. The solution can be found in the online material referenced at the beginning of this section.

This shows that any two sets of initial conditions A and B will have identical values for $\dot{\theta}$ but different values for $\ddot{\theta}$ at t_A and $t_B = t_A + \delta$, respectively. Moreover, it is easy to find sets of initial conditions for which $\delta \neq 0$, and choose a value for $\varepsilon \neq 0$. This shows that $\dot{\theta}$ is not Markov. □

4 Non-Markov Second Derivative of θ

Finally, we show that $\ddot{\theta}$ is not Markov, either:

Lemma 4.1 ($\ddot{\theta}$ is not Markov)**.** *For $\ddot{\theta}_A(t)$ governed by initial conditions $A = \{\nu_A, \phi_A\}$ and agent trajectory $a(t)$, there exist initial conditions $B = \{\nu_B, \phi_B\}$, $A \neq B$, time steps t_A, t_B, and an agent trajectory $a(t)$ such that (i) $\ddot{\theta}_A(t_A) = \ddot{\theta}_B(t_B)$ but (ii) $\dddot{\theta}_A(t_A) \neq \dddot{\theta}_B(t_B)$.*

Proof. The proof is carried out equivalently as for $\dot{\theta}$. We begin by computing the third derivative of θ:

$$\dddot{\theta}(t) = \frac{3\nu g^3 \sin^2(\phi)}{(\nu \sin(\phi) - gt)^4 \cos(\phi)}. \tag{8}$$

We can then find an expression for δ by solving $\dddot{\theta}_A(t_A) = \dddot{\theta}_B(t_B)$ for δ. Again, we omit writing out δ explicitly and state that it is possible to find sets of initial conditions for which $\delta \neq 0$ and choose $\varepsilon \neq 0$. Thus $\ddot{\theta}$ is not Markov. □

Proof. Taken together, the three Lemmas 2.1-4.1 prove Theorem 1.1. □

References

1. Meurer A, Smith CP, Paprocki M, Čertík O, Kirpichev SB, Rocklin M, et al. SymPy: symbolic computing in Python. PeerJ Computer Science. 2017;3:e103.
