# Supplementary material for: No free lunch in ball catching: A comparison of Cartesian and angular representations for control
Source: PLoS One. 2018 Jun 14;13(6):e0197803. doi: 10.1371/journal.pone.0197803 (PMC6002113; doi:10.1371/journal.pone.0197803)
Supplement: S2 Text — Formal explanation of the moving averaging computation and parameters for LQG, iLQG and MPC control strategies used in Section 4.4. (PDF) [file pone.0197803.s003.pdf]

No Free Lunch in Ball Catching: A Comparison of Cartesian and Angular Representations for Control Supplementary Material (S2 Text). Comparison of Cartesian and Angular Control: Supplementary Material for Experiments

Contents

1 COV-IO and COV-OAC: Computation of Moving Average Filter 1

2 (i)LQG: Parameters 2
2.1 Dynamics (Ideal Case) . . . 2
2.2 Dynamics (Drag Case) . . . 3
2.3 Observation Model . . . 3
2.4 LQR and iLQR Cost Function . . . 4
2.5 Kalman Filter Parameters . . . 4

3 Model-Predictive Control in Belief Space (MPC): Parameters 5

1 COV-IO and COV-OAC: Computation of Moving Average Filter

To increase the robustness of COV-IO and COV-OAC, we filter both the estimate $\dot{\theta}_{\text{ref}}$ as well as the current reference velocity $\dot{\theta}$. To that end, we use a (moving) average with window sizes $h_{\dot{\theta}_{\text{ref}}}$ and $h_{\dot{\theta}}$:

$$\dot{\theta}_{\text{ref}}^{\text{COV-IO}} = \frac{1}{H_{\dot{\theta}_{\text{ref}}}} \sum_{i=0}^{H_{\dot{\theta}_{\text{ref}}}} \dot{\theta}(i\Delta t), \tag{1}$$

$$\dot{\theta}^{\text{COV-IO}}(t) = \frac{1}{H_{\dot{\theta}}} \sum_{i=0}^{H_{\dot{\theta}}} \dot{\theta}(t - i\Delta t). \tag{2}$$

where $H_{\dot{\theta}_{\text{ref}}} = h_{\dot{\theta}_{\text{ref}}} \Delta t^{-1}$ and $H_{\dot{\theta}} = h_{\dot{\theta}} \Delta t^{-1}$ are the discrete time steps corresponding to the window sizes given in seconds.

We apply the same type of filtering for COV-OAC, where we denote the window sizes by $h_{\dot{\theta}}, h_{\dot{\theta}_{\text{ref}}}$.

2 (i)LQG: Parameters

In our implementation we extend the Cartesian state representation to include the acceleration:

$$\mathbf{x} = [b_x, \dot{b}_x, \ddot{b}_x, b_y, \dot{b}_y, \ddot{b}_y, b_z, \dot{b}_z, a_x, \ddot{a}_x, \ddot{a}_x, a_z, \ddot{a}_z, \ddot{a}_z]^T. \tag{3}$$

This allows us to directly incorporate gravity g into the model (see $\mathbf{e}$).

2.1 Dynamics (Ideal Case)

We assume discrete-time linear system dynamics of the following form:

$$\mathbf{x}(t+1) = \mathbf{A}\mathbf{x}(t) + \mathbf{B}\mathbf{u}(t) + \mathbf{e} + \varepsilon_t \tag{4}$$

where $\mathbf{A} \in \mathbb{R}^{n\times n}$, $\mathbf{B} \in \mathbb{R}^{n\times m}$ and $\mathbf{e} \in \mathbb{R}^n$ are the state-dependent, action-dependent and constant system dynamics matrices, and $\varepsilon_t \sim \mathcal{N}(\mathbf{0}, \mathbf{\Sigma}_\varepsilon)$ is a multivariate Gaussian with zero-mean and covariance $\mathbf{\Sigma}_\varepsilon$.

Given a time step Δt, the dynamics for ball catching in the ideal case are given by:

$$\mathbf{A} = \begin{pmatrix}
1 & \Delta t & \frac{1}{2}\Delta t^2 & 0 & 0 & 0 & 0 & 0 & 0 & 0 & 0 & 0 & 0 & 0 & 0 \\
0 & 1 & \Delta t & 0 & 0 & 0 & 0 & 0 & 0 & 0 & 0 & 0 & 0 & 0 & 0 \\
0 & 0 & 0 & 0 & 0 & 0 & 0 & 0 & 0 & 0 & 0 & 0 & 0 & 0 & 0 \\
0 & 0 & 0 & 1 & \Delta t & \frac{1}{2}\Delta t^2 & 0 & 0 & 0 & 0 & 0 & 0 & 0 & 0 & 0 \\
0 & 0 & 0 & 0 & 1 & \Delta t & 0 & 0 & 0 & 0 & 0 & 0 & 0 & 0 & 0 \\
0 & 0 & 0 & 0 & 0 & 0 & 0 & 0 & 0 & 0 & 0 & 0 & 0 & 0 & 0 \\
0 & 0 & 0 & 0 & 0 & 0 & 1 & \Delta t & \frac{1}{2}\Delta t^2 & 0 & 0 & 0 & 0 & 0 & 0 \\
0 & 0 & 0 & 0 & 0 & 0 & 0 & 1 & \Delta t & 0 & 0 & 0 & 0 & 0 & 0 \\
0 & 0 & 0 & 0 & 0 & 0 & 0 & 0 & 0 & 0 & 0 & 0 & 0 & 0 & 0 \\
0 & 0 & 0 & 0 & 0 & 0 & 0 & 0 & 0 & 1 & \Delta t & 0 & 0 & 0 & 0 \\
0 & 0 & 0 & 0 & 0 & 0 & 0 & 0 & 0 & 0 & 1 & 0 & 0 & 0 & 0 \\
0 & 0 & 0 & 0 & 0 & 0 & 0 & 0 & 0 & 0 & 0 & 0 & 0 & 0 & 0 \\
0 & 0 & 0 & 0 & 0 & 0 & 0 & 0 & 0 & 0 & 0 & 0 & 1 & \Delta t & 0 \\
0 & 0 & 0 & 0 & 0 & 0 & 0 & 0 & 0 & 0 & 0 & 0 & 0 & 1 & 0 \\
0 & 0 & 0 & 0 & 0 & 0 & 0 & 0 & 0 & 0 & 0 & 0 & 0 & 0 & 0
\end{pmatrix}, \tag{5}$$

$$\mathbf{B} = \begin{pmatrix}
0 & 0 & 0 & 0 & 0 & 0 & 0 & 0 & 0 & \frac{1}{2}\Delta t^2 & \Delta t & 0 & 0 & 0 & 0 \\
0 & 0 & 0 & 0 & 0 & 0 & 0 & 0 & 0 & 0 & 0 & 0 & \frac{1}{2}\Delta t^2 & \Delta t & 0
\end{pmatrix}^T \tag{6}$$

$$\mathbf{e} = \begin{pmatrix} 0 & 0 & 0 & 0 & 0 & -g & 0 & 0 & 0 & 0 & 0 & 0 & 0 & 0 & 0 \end{pmatrix}^T, \tag{7}$$

where $g = 9.81$ denotes the average magnitude of gravity on earth.

2.2 Dynamics (Drag Case)

When adding drag to the ball trajectory, the process model becomes nonlinear. We thus need to compute the linearization of the state dynamics, the Jacobian $\mathbf{J}_T$:

$$\mathbf{J}_T(\dot{\mathbf{b}}) = \begin{pmatrix}
1 & \Delta t & \frac{1}{2}\Delta t^2 & 0 & 0 & 0 & 0 & 0 & 0 & 0 & 0 & 0 & 0 & 0 & 0 \\
0 & 1 & \Delta t & 0 & 0 & 0 & 0 & 0 & 0 & 0 & 0 & 0 & 0 & 0 & 0 \\
0 & -\frac{2\rho c A c_d}{m_b}\dot{b}_x & 0 & 0 & 0 & 0 & 0 & 0 & 0 & 0 & 0 & 0 & 0 & 0 & 0 \\
0 & 0 & 0 & 1 & \Delta t & \frac{1}{2}\Delta t^2 & 0 & 0 & 0 & 0 & 0 & 0 & 0 & 0 & 0 \\
0 & 0 & 0 & 0 & 1 & \Delta t & 0 & 0 & 0 & 0 & 0 & 0 & 0 & 0 & 0 \\
0 & 0 & 0 & 0 & -\frac{2\rho c A c_d}{m_b}\dot{b}_y & 0 & 0 & 0 & 0 & 0 & 0 & 0 & 0 & 0 & 0 \\
0 & 0 & 0 & 0 & 0 & 0 & 1 & \Delta t & \frac{1}{2}\Delta t^2 & 0 & 0 & 0 & 0 & 0 & 0 \\
0 & 0 & 0 & 0 & 0 & 0 & 0 & 1 & \Delta t & 0 & 0 & 0 & 0 & 0 & 0 \\
0 & 0 & 0 & 0 & 0 & 0 & 0 & -\frac{2\rho c A c_d}{m_b}\dot{b}_z & 0 & 0 & 0 & 0 & 0 & 0 & 0 \\
0 & 0 & 0 & 0 & 0 & 0 & 0 & 0 & 0 & 1 & \Delta t & 0 & 0 & 0 & 0 \\
0 & 0 & 0 & 0 & 0 & 0 & 0 & 0 & 0 & 0 & 1 & 0 & 0 & 0 & 0 \\
0 & 0 & 0 & 0 & 0 & 0 & 0 & 0 & 0 & 0 & 0 & 1 & 0 & 0 & 0 \\
0 & 0 & 0 & 0 & 0 & 0 & 0 & 0 & 0 & 0 & 0 & 0 & 1 & \Delta t & 0 \\
0 & 0 & 0 & 0 & 0 & 0 & 0 & 0 & 0 & 0 & 0 & 0 & 0 & 1 & 0 \\
0 & 0 & 0 & 0 & 0 & 0 & 0 & 0 & 0 & 0 & 0 & 0 & 0 & 0 & 1
\end{pmatrix} \quad (8)$$

We assume air density $\rho = 1.293 \frac{\text{kg}}{\text{m}^3}$, frontal area $A = \pi r^2$. For a baseball we assume the following dynamic parameters [1]: ball radius $r = 0.0366\,\text{m}$, mass $m_b = 0.15\,\text{kg}$ and drag coefficient $c_w = 0.5$. For a soccer ball, we assume: ball radius $r = 0.11\,\text{m}$, mass $m_b = 0.4\,\text{kg}$ and drag coefficient $c_w = 0.3$ [2].

2.3 Observation Model

We assume a linear *observation model* which maps the state $\mathbf{x}(t)$ to an observation $\mathbf{z}(t)$:

$$\mathbf{z}(t) = \mathbf{C}\mathbf{x}(t) + \mathbf{c} + \delta_t, \quad (9)$$

where $\mathbf{C} \in \mathbb{R}^{q \times n}$ are the state-dependent and $\mathbf{c} \in \mathbb{R}^q$ the constant measurement matrix, and $\delta_t \sim \mathcal{N}(\mathbf{0}, \mathbf{\Sigma}_\delta)$ is a multivariate Gaussian with zero-mean and covariance $\mathbf{\Sigma}_\delta$.

In both the ideal and drag case, the observation model for ball catching is defined by the following quantities.

$$\mathbf{C} = \begin{pmatrix}
1 & 0 & 0 & 0 & 0 & 0 & 0 & 0 & 0 & 0 & 0 & 0 & 0 & 0 & 0 \\
0 & 0 & 0 & 1 & 0 & 0 & 0 & 0 & 0 & 0 & 0 & 0 & 0 & 0 & 0 \\
0 & 0 & 0 & 0 & 0 & 0 & 1 & 0 & 0 & 0 & 0 & 0 & 0 & 0 & 0 \\
0 & 0 & 0 & 0 & 0 & 0 & 0 & 0 & 0 & 1 & 0 & 0 & 0 & 0 & 0 \\
0 & 0 & 0 & 0 & 0 & 0 & 0 & 0 & 0 & 0 & 1 & 0 & 0 & 0 & 0 \\
0 & 0 & 0 & 0 & 0 & 0 & 0 & 0 & 0 & 0 & 0 & 0 & 1 & 0 & 0 \\
0 & 0 & 0 & 0 & 0 & 0 & 0 & 0 & 0 & 0 & 0 & 0 & 0 & 1 & 0
\end{pmatrix}. \quad (10)$$

$$\mathbf{c} = \mathbf{0}. \quad (11)$$

2.4 LQR and iLQR Cost Function

We assume a *quadratic finite-horizon cost function* of the following form:

$$\mathcal{L}_{\mathrm{LQG}} = \frac{1}{2}\mathbf{x}^T(T)\mathbf{Q}_T\mathbf{x}(T) + \frac{1}{2}\sum_{t=0}^{T-1}\mathbf{x}^T(t)\mathbf{Q}_t\mathbf{x}(t) + \mathbf{u}^T(t)\mathbf{R}_t\mathbf{u}(t), \tag{12}$$

with terminal state cost matrix $\mathbf{Q}_T \in \mathbb{R}^{n\times n}$, running state cost matrix $\mathbf{Q}_t \in \mathbb{R}^{n\times n}$ and control input cost matrix $\mathbf{R}_t \in \mathbb{R}^{m\times m}$. The matrices $\mathbf{Q}_T$, $\mathbf{Q}_t$ are required to be real symmetric positive semi-definite matrices and $\mathbf{R}_t$ to be a real symmetric positive definite matrix. The *time horizon* T must be greater than 0 and finite.

We define the terminal cost matrix for ball catching as follows.

$$Q_T = 2\begin{pmatrix}
w_\mathrm{d} & 0 & 0 & 0 & 0 & 0 & 0 & 0 & 0 & -w_\mathrm{d} & 0 & 0 & 0 & 0 & 0\\
0 & 0 & 0 & 0 & 0 & 0 & 0 & 0 & 0 & 0 & 0 & 0 & 0 & 0 & 0\\
0 & 0 & 0 & 0 & 0 & 0 & 0 & 0 & 0 & 0 & 0 & 0 & 0 & 0 & 0\\
0 & 0 & 0 & 0 & 0 & 0 & 0 & 0 & 0 & 0 & 0 & 0 & 0 & 0 & 0\\
0 & 0 & 0 & 0 & 0 & 0 & 0 & 0 & 0 & 0 & 0 & 0 & 0 & 0 & 0\\
0 & 0 & 0 & 0 & 0 & 0 & 0 & 0 & 0 & 0 & 0 & 0 & 0 & 0 & 0\\
0 & 0 & 0 & 0 & 0 & 0 & w_\mathrm{d} & 0 & 0 & 0 & 0 & 0 & -w_\mathrm{d} & 0 & 0\\
0 & 0 & 0 & 0 & 0 & 0 & 0 & 0 & 0 & 0 & 0 & 0 & 0 & 0 & 0\\
0 & 0 & 0 & 0 & 0 & 0 & 0 & 0 & 0 & 0 & 0 & 0 & 0 & 0 & 0\\
-w_\mathrm{d} & 0 & 0 & 0 & 0 & 0 & 0 & 0 & 0 & w_\mathrm{d} & 0 & 0 & 0 & 0 & 0\\
0 & 0 & 0 & 0 & 0 & 0 & 0 & 0 & 0 & 0 & w_\mathrm{d} & 0 & 0 & 0 & 0\\
0 & 0 & 0 & 0 & 0 & 0 & 0 & 0 & 0 & 0 & 0 & 0 & 0 & 0 & 0\\
0 & 0 & 0 & 0 & 0 & 0 & -w_\mathrm{d} & 0 & 0 & 0 & 0 & 0 & w_\mathrm{d} & 0 & 0\\
0 & 0 & 0 & 0 & 0 & 0 & 0 & 0 & 0 & 0 & 0 & 0 & 0 & 0 & 0\\
0 & 0 & 0 & 0 & 0 & 0 & 0 & 0 & 0 & 0 & 0 & 0 & 0 & 0 & 0
\end{pmatrix}, \tag{13}$$

where we use the abbreviation $w_\mathrm{d} = w_{\text{terminal distance}}$.

The running state cost Q_t is the $N \times N$ zero matrix, and the running control cost is given by

$$R_t = w_{\text{control effort}}\mathbf{I}_M. \tag{14}$$

with $\mathbf{I}_M$ denoting the $M \times M$ identity matrix.

2.5 Kalman Filter Parameters

The Kalman filter uses the same dynamics model as LQR. Additionally, we need to provide the following quantities.

We set the initial state covariance as:

$$\mathbf{\Sigma}_0 = \mathrm{diag}(1, 600, 10^{-15}, 1, 600, 10^{-15}, 1, 600, 10^{-15}, 0.1, 6, 10^{-15}, 0.1, 6, 10^{-15}), \tag{15}$$

where diag denotes a diagonal matrix.

The process noise is given by:

$$\mathbf{V} = \mathrm{diag}(10^{-5}, 6\times10^{-4}, 10^{-5}, 10^{-5}, 6\times10^{-4}, 10^{-5}, 10^{-5}, 6\times10^{-4}, 10^{-5}, \tag{16}$$

$$10^{-5}, 6\times10^{-4}, 10^{-5}, 10^{-5}, 6\times10^{-4}, 10^{-5}). \tag{17}$$

The measurement noise is given by:

$$\mathbf{W} = \text{diag}(10^{-5}, 10^{-5}, 10^{-5}, 10^{-5}, 6 \times 10^{-4}, 10^{-5}, 6 \times 10^{-4}). \quad (18)$$

The parameters for the Kalman filter were determined in preliminary experiments to be optimal for the average case. We use the same parameters for the linear as well as the extended Kalman filter.

3 Model-Predictive Control in Belief Space (MPC): Parameters

For the MPC control strategy [3], we ran preliminary experiments to identify the best set of parameters. We used the implementation provided by the authors:
`https://github.com/b4be1/easy_catch`.

To guarantee full observability, we always set the observation noise related to the location of the ball in the agent's field of view to $\sigma_{\min} = \sigma_{\max} = 10^{-15}$, where $\sigma_{\min}$ denotes the observation noise when looking directly at the ball and $\sigma_{\max}$ when the ball is at $\geq 90°$ from the gaze direction. This effectively results in a full view. We varied the forward and backward running speeds F_1, F_2 and the scalar weight multiplied with the system covariance M. For all other parameters, we used the same settings as used by the authors, including the internal model which is equivalent to the one used by (i)LQG without drag.

We tested the following combinations of parameters:

Description	F_1	F_2	M
Best	7.5	7.5	10^{-15}
Uncertain	7.5	7.5	10^{-3}
Default	7.5	2.5	10^{-3}
Slower	5	5	10^{-15}
Slower, uncertain	5	5	10^{-3}

Table A. Parameter settings evaluated for the strategy proposed by [3].

For comparison: the setting used by [3] is $F_1 = 7.5$, $F_2 = 2.5$, $M = 10^{-3}$, $\sigma_{\min} = 0.01$, $\sigma_{\max} = 1$.

References

1. NASA. Drag on a baseball; 2015. `https://www.grc.nasa.gov/WWW/k-12/airplane/balldrag.html`.

2. NASA. Drag on a soccer ball; 2015. `https://www.grc.nasa.gov/www/k-12/airplane/socdrag.html`.

3. Belousov B, Neumann G, Rothkopf CA, Peters J. Catching heuristics are optimal control policies. In: Advances in Neural Information Processing Systems (NIPS). Barcelona, Spain; 2016. p. 1426–1434.
