# Supplementary material for: No free lunch in ball catching: A comparison of Cartesian and angular representations for control
Source: PLoS One. 2018 Jun 14;13(6):e0197803. doi: 10.1371/journal.pone.0197803 (PMC6002113; doi:10.1371/journal.pone.0197803)
Supplement: S3 Text — Detailed explanation of camera simulation used in Section 5, as well as how the baseline policies in this section are computed and compared to the learned policies. (PDF) [file pone.0197803.s004.pdf]

No Free Lunch in Ball Catching: A Comparison of Cartesian and Angular Representations for Control
Supplementary Material (S3 Text)
On the Optimality of Chapman's Strategy: Supplementary Material for Experiments

Contents

1 Camera Model **1**

2 Baseline: COV-IO and COV-OAC **3**

2.1 Comparison of Baseline and CMA-ES Policies 3

1 Camera Model

In order to perform learning on raw sensor data, we simulate a one-dimensional camera sensor. To mimic characteristics of real sensors, we assume that the camera has a limited angle of view and may distort the image at the borders (common vision sensors exhibit an angle of view below 90°). To provide the robot with a full 180° view (required to ensure full observability) we arrange two pinhole camera models c_1 and c_2 each with an angle of view of 90°, as shown in Figure A. At time step t, each camera $c_i \in \{1, 2\}$ outputs one image $\tilde{\mathbf{o}}^{c_i}(\mathrm{t})$. By concatenating the two camera images, we obtain the *raw observation* $\tilde{\mathbf{o}}(\mathrm{t})$:

$$\tilde{\mathbf{o}}(t) = \tilde{\mathbf{o}}^{c_1}(t) \oplus \tilde{\mathbf{o}}^{c_2}(t), \tag{1}$$

where $\oplus$ denotes the concatenation operator.

Camera Arrangement The first camera c_1 is tilted by $\eta^{c_1} = \frac{\pi}{4}$, thus covering the range from $\gamma^{c_1}_{\text{min}} = 0$ to $\gamma^{c_1}_{\text{max}} = \frac{\pi}{2}$, and the second camera c_2 is tilted by $\eta^{c_1} = \frac{3\pi}{4}$, covering the remaining area $\gamma^{c_2}_{\text{min}} = \frac{\pi}{2}$ to $\gamma^{c_2}_{\text{max}} = \pi$. Note, however, that the pixels do not correspond to angles, but the image is distorted at the borders of each camera due to the projection.

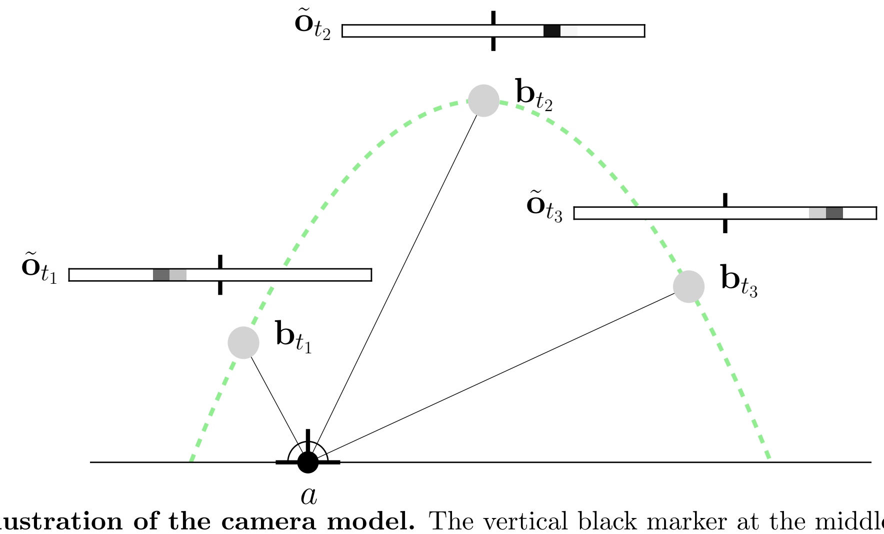

Fig A. Illustration of the camera model. The vertical black marker at the middle of each observation indicates that the full image is computed by concatenating the images of two cameras with a 90° angle of view.

Resolution We denote by ρ the resolution of both cameras c_i. The number of pixels N^{c_i} of camera c_i then computes as (omitting c_i to increase readability)

$$N = \left\lceil \frac{\tan(\gamma_{\text{max}} - \eta) - \tan(\gamma_{\text{min}} - \eta)}{\rho} + 1 \right\rceil, \tag{2}$$

where ρ denotes the resolution of the camera.

Image Projection To compute the value of a pixel $\tilde{\mathbf{o}}_i$ in the image, we project the ball on the image as follows. First we compute α by using the center of the ball as its coordinate. Additionally, we need to take the ball's radius r into account to compute the angles α_{left} and α_{right} for left and right border of the ball, respectively:

$$\alpha_{\text{left}} = \arctan \frac{b_y - r\cos(-\alpha)}{a_x - b_x - r\sin(-\alpha)}, \tag{3}$$

$$\alpha_{\text{right}} = \arctan \frac{b_y + r\cos(-\alpha)}{a_x - b_x + r\sin(-\alpha)}. \tag{4}$$

where b_x, b_y, a_x, a_y are the x- and y-coordinates of the ball and agent, respectively. Using these angles we then compute a *hit map* $h : \{1, \ldots, N\} \to \{0, 1\}$ indicating onto which pixels the ball is projected:

$$h(k) = \begin{cases} 1 & \text{if} \quad \alpha_{\text{left}} \leq \eta + \arctan v_k \leq \alpha_{\text{right}} \\ 0 & \text{else} \end{cases} \tag{5}$$

with $v_k = \tan(\gamma_{\text{min}} - \eta) + k\lambda$ being the (tangent of) the angle that the k-th index of the image corresponds to. Finally, we generate the raw camera image $\tilde{\mathbf{o}}^{c_i}$ by smoothing the hit map. To do

this we propagate a hit to adjacent pixels by computing for every pixel how much it has been hit and assigning this value to the final image pixel:

$$\tilde{\mathbf{o}}^{c_i}(k) = \max\left(0, \frac{1 - |v - v_k|}{\rho}\right), \tag{6}$$

with $v = \tan(\alpha - \eta)$ being the tangent of angle corresponding to the ball's center.

2 Baseline: COV-IO and COV-OAC

To implement the baselines, we apply supervised learning to learn a predictor $\Omega(\mathbf{o}) = \mathbf{w}_\Omega^T \mathbf{o}$ mapping from observation $\tilde{\mathbf{o}}$ to angle θ. We train Ω by collecting a training set of ball trajectories without agent motion. We collect five trajectories each with a different agent starting position $D \in \{-15, -7.5, 0, 7.5, 15\}$. This results in a large set of $(\tilde{\mathbf{o}}_t, \theta_t)$-pairs, consisting of raw observations and known value of the true tangent. We then compute a ridge regression from $\tilde{\mathbf{o}}_t$ to θ_t with regularization $\alpha = 10^{-15}$ and without bias term, obtaining a weight vector $\mathbf{w}_\Omega$. Note that because Ω is linear it can also be used to compute the mapping from $\dot{\tilde{\mathbf{o}}}$ to $\dot{\theta}$.

We can then define the following two strategies:

COV-IO$^\mathbf{o}$ We use the augmented observation *current and initial*: $\pi(\dot{\mathbf{o}}_t) = \pi(\dot{\tilde{\mathbf{o}}}_t \oplus \dot{\tilde{\mathbf{o}}}_\text{init}) = v_\Omega \left(\mathbf{w}_\Omega^T \dot{\tilde{\mathbf{o}}}_t - \mathbf{w}_\Omega^T \dot{\tilde{\mathbf{o}}}_\text{init}\right)$ with $v_\Omega = 10^{10}$ being a control gain factor. Intuitively, assuming $\mathbf{w}_\Omega^T \dot{\tilde{\mathbf{o}}}_t \propto \dot{\theta}_t$, this strategy implements COV-IO, with v_Ω driving the policy output to saturation and thus effectively implementing bang-bang control.

COV-OAC$^\mathbf{o}$ We use the augmented observation *current and delayed*: $\pi(\dot{\mathbf{o}}_t) = \pi(\dot{\tilde{\mathbf{o}}}_t \oplus \dot{\tilde{\mathbf{o}}}_{t-t_\text{delay}}) = v_\Omega \left(\mathbf{w}_\Omega^T \dot{\tilde{\mathbf{o}}}_t - \mathbf{w}_\Omega^T \dot{\tilde{\mathbf{o}}}_{t-t_\text{delay}}\right)$. Applying a similar line of reasoning as before, we see that this strategy approximates COV-OAC.

2.1 Comparison of Baseline and CMA-ES Policies

We compare the baseline and the CMA-ES strategies as follows. We only consider the pairs COV-IO$^\mathbf{o}$ vs. CMA-ES applied to $\mathbf{o} = \dot{\tilde{\mathbf{o}}}_t \oplus \dot{\tilde{\mathbf{o}}}_{t_\text{init}}$ and COV-OAC$^\mathbf{o}$ vs. CMA-ES applied to $\mathbf{o} = \dot{\tilde{\mathbf{o}}}_t \oplus \dot{\tilde{\mathbf{o}}}_{t-t_\text{delay}}$.

We then employ two types of comparisons. First, we compare the outputs of both policies applied to the data COV-IO$^\mathbf{o}$ and COV-OAC$^\mathbf{o}$ (or more precisely: Ω) were trained on. Since the baseline policies implement bang-bang controllers we assume the CMA-ES policies do so too. Hence, we discretize the output of both policies $y \in \{-1, 0, 1\}$ and use a zero-one loss:

$$\mathcal{L}(\Omega, \{(\mathbf{o}^{(i)}, y^{(i)})\}_{i=1,\dots,N}) = \frac{1}{N} \sum_{i=0}^{N} (1 - \mathbb{1}\{\Omega(\mathbf{o}^{(i)}) = y^{(i)}\}), \tag{7}$$

to compare the outputs ($\mathbb{1}\{\,\cdot\,\}$ denotes the indicator function which evaluates to 1 if the expression inside the brackets is true and to 0 otherwise). This results in the values in the third column of Table A.

Second, we compare the weights. Again, we assume a bang-bang controller and thus only look at the pixel-wise weight vector, but flip the sign of the CMA-ES policy vector according to the sign of

Baseline	CMA-ES Augmentation	Similarity: Predictions	Similarity: Weights
COV-IO$^{\mathbf{o}}$	$\mathbf{o} = \dot{\mathbf{o}}_t \oplus \dot{\mathbf{o}}_{t_{\text{init}}}$	0.898	0.997 (dimensions: $\{12, 13, 14, 15, 16, 17, 33, 34\}$)
COV-OAC$^{\mathbf{o}}$	$\mathbf{o} = \dot{\mathbf{o}}_t \oplus \dot{\mathbf{o}}_{t-t_{\text{delay}}}$	0.77	0.916 (dimensions: $\{12, 13, 14, 15, 16, 17, 29, 30, 31, 32, 33, 34\}$)

Table A. Results for different metrics used to compare the baseline policies with the policies learned by CMA-ES.

v_Ω. We then compute a histogram over the absolute value of all pixels of the (augmented) observed images in the training data. We then compute the value of the most active pixel dimension and disregard all image dimensions that lie below 10% of this value. This results in the dimensions listed in the forth column of Table A. Note that the dimensions ≤ 17 pertain to $\dot{\mathbf{o}}_t$ and the remaining ones to $\dot{\mathbf{o}}_{t_{\text{init}}}$ or $\dot{\mathbf{o}}_{t-t_{\text{delay}}}$. We then compute Pearson's correlation coefficient $\bar{\rho}$ comparing the two weight vectors, restricted to the subset of dimensions. This results in the values in the last column of Table A. Note that Pearson's correlation implicitly performs a linear regression between the two weight vectors which includes a bias term. The reason why we need a bias term here is because the disregarded dimensions are not completely zero and would thus contribute to the overall result of each policy.
