# Supplementary material for: No free lunch in ball catching: A comparison of Cartesian and angular representations for control
Source: PLoS One. 2018 Jun 14;13(6):e0197803. doi: 10.1371/journal.pone.0197803 (PMC6002113; doi:10.1371/journal.pone.0197803)
Supplement: S4 Text — A concise and formal introduction to Linear-Quadratic Gaussian control (LQG), including linear-quadratic regulators, Kalman filters and its nonlinear extensions iterative LQG (iLQG) and extended Kalman filters, respectively. (PDF) [file pone.0197803.s005.pdf]

No Free Lunch in Ball Catching: A Comparison of Cartesian and Angular Representations for Control
Supplementary Material (S4 Text)
Introduction to Linear-Quadratic Gaussian Control

Contents

1 Introduction **1**

2 Linear-Quadratic Regulator (LQR) **2**

3 Iterative LQR (iLQR) **2**

4 Kalman Filter **3**

5 Extended Kalman Filter **3**

1 Introduction

Linear-quadratic Gaussian control (LQG) consists of two components: a *linear-quadratic regulator (LQR)* that computes the control output given a state and a *Kalman filter* that estimates the current state from the current observation and previous states. There exist various variants of LQG that differ with respect to whether they consider *finite or infinite time horizon*, *discrete or continuous time* and *stationary or non-stationary dynamics*. We will use finite-horizon discrete-time LQG with stationary dynamics in our application and present it in the following.

Both assume a linear transition function T, which we call the *system dynamics* or *process model*:

$$\mathbf{x}(t+1) = T(\mathbf{x}(t), \mathbf{u}(t)) \tag{1}$$

$$= \mathbf{A}\mathbf{x}(t) + \mathbf{B}\mathbf{u}(t) + \mathbf{e} + \varepsilon_t \tag{2}$$

where $\mathbf{A} \in \mathbb{R}^{n \times n}$, $\mathbf{B} \in \mathbb{R}^{n \times m}$ and $\mathbf{e} \in \mathbb{R}^n$ are the state-dependent, action-dependent and constant system dynamics matrices, and $\varepsilon_t \sim \mathcal{N}(\mathbf{0}, \mathbf{\Sigma}_\varepsilon)$ is a multivariate Gaussian with zero-mean and covariance $\mathbf{\Sigma}_\varepsilon$.

Moreover, we define a *measurement model* which maps the state $\mathbf{x}(t)$ to an observation $\mathbf{z}(t)$:

$$\mathbf{z}(t) = \mathbf{C}\mathbf{x}(t) + \mathbf{c} + \delta_t, \tag{3}$$

where $\mathbf{C} \in \mathbb{R}^{q\times n}$ are the state-dependent and $\mathbf{c} \in \mathbb{R}^{q}$ the constant measurement matrix, and $\delta_t \sim \mathcal{N}(\mathbf{0}, \mathbf{\Sigma}_\delta)$ is a multivariate Gaussian with zero-mean and covariance $\mathbf{\Sigma}_\delta$.

The goal of LQG is to minimize a *quadratic finite-horizon cost function* $\mathcal{L}_{\text{LQG}}$ of the following form:

$$\mathcal{L}_{\text{LQG}} = \frac{1}{2}\mathbf{x}^T(T)\mathbf{Q}_T\mathbf{x}(T) + \frac{1}{2}\sum_{t=0}^{T-1}\mathbf{x}^T(t)\mathbf{Q}_t\mathbf{x}(t) + \mathbf{u}^T(t)\mathbf{R}_t\mathbf{u}(t), \tag{4}$$

with terminal state cost matrix $\mathbf{Q}_T \in \mathbb{R}^{n\times n}$, running state cost matrix $\mathbf{Q}_t \in \mathbb{R}^{n\times n}$ and control input cost matrix $\mathbf{R}_t \in \mathbb{R}^{m\times m}$. The matrices $\mathbf{Q}_T$, $\mathbf{Q}_t$ are required to be real symmetric positive semi-definite matrices and $\mathbf{R}_t$ to be a real symmetric positive definite matrix. The *time horizon* T must be greater than 0 and finite.

2 Linear-Quadratic Regulator (LQR)

We now show how to derive optimal control outputs $\mathbf{u}$ for the system in Eq. (1) with respect to the cost function $\mathcal{L}$ from Equation 4. For LQR problems the optimal control output can be expressed as

$$\mathbf{u}(t) = \mathbf{F}_t\mathbf{x}(t) + \mathbf{f}_t, \tag{5}$$

where

$$\mathbf{F}_t = -\mathbf{B}^T\mathbf{P}_t\mathbf{B} + \mathbf{R}^{-1}\mathbf{B}^T\mathbf{P}_t\mathbf{A} \tag{6}$$

$$\mathbf{f}_t = -\mathbf{B}^T\mathbf{P}_t\mathbf{B} + \mathbf{R}^{-1}\mathbf{B}^T(\mathbf{p}_t + \mathbf{P}_t\mathbf{e}). \tag{7}$$

The terms $\{(\mathbf{P}_t, \mathbf{p}_t)\}_{t\in\{0,\ldots,N-1\}}$ can be calculated analytically using dynamic programming, by applying the *discrete-time finite horizon Riccati equation* iteratively backwards in time:

$$\mathbf{P}_{t-1} = \mathbf{A}^T\mathbf{P}_t\mathbf{A} + \mathbf{Q} + (\mathbf{B}^T\mathbf{P}_t\mathbf{A})^T\mathbf{P}_t \tag{8}$$

$$\mathbf{p}_{t-1} = \mathbf{A}^T(\mathbf{p}_t + \mathbf{P}_t\mathbf{e}) + (\mathbf{B}^T\mathbf{P}_t\mathbf{A})^T\mathbf{p}_t \tag{9}$$

from initial condition $\mathbf{P}_N = \mathbf{Q}_T$ and $\mathbf{p}_N = \mathbf{0}$. For a time interval of length T seconds and time constant Δt this procedure results in a set of $N = \lfloor \frac{T}{\Delta t} \rfloor$ control gain parameters $(\mathbf{F}_t, \mathbf{f}_t)$.

Further note that LQR results in a deterministic policy despite the assumption of a dynamics model that is perturbed by Gaussian noise; this is indeed an intrinsic feature of the LQG formulation that can be seen when deriving the optimal control gains [1].

3 Iterative LQR (iLQR)

LQR is only applicable to problems that exhibit linear system dynamics and a quadratic cost function. To overcome this problem, [2] proposed the *iterative LQR (iLQR)* method which is applicable to nonlinear system dynamics (and nonlinear cost, too; see [3]), under the assumption that dynamics (and cost) are differentiable.

The method starts with a *nominal control sequence* $U^0 = \{\mathbf{u}_0, \ldots, \mathbf{u}_{T-1}\}$ (usually $\mathbf{u}_k = 0$ for $k = 0, \ldots T-1$) and computes an open-loop forward pass using the (non-linear) system dynamics to obtain the *nominal trajectory* $X^0 = \{\mathbf{x}_0, \ldots, \mathbf{u}_T\}$. It then computes an improved control sequence U^1 by linearizing the nonlinear dynamics around X^0, U^0, yielding the (time-varying) linearized dynamics $\mathbf{A}_t$, $\mathbf{B}_t$ and $\mathbf{e}_t$ and using them to solve a standard LQR problem. This process is repeated until the control sequence U does not change anymore.

4 Kalman Filter

The LQR controller assumes that the agent can directly observe the current state $\mathbf{x}$. However, the agent only has access to an observation $\mathbf{z}$. If we assume the relationship between $\mathbf{x}$ and $\mathbf{z}$ to be given by the measurement model (Eq. 3) and we have access to the process model (Eq. 1), we can apply *Bayesian filtering* (or *recursive estimation*) to infer the state from observations. A Bayes filter maintains a probability distribution over the state and updates it in every time step given the new observation, the previous state distribution and the last control signal. If process and measurement model are linear, we can apply a *Kalman filter* which models the state distribution by a Gaussian with mean $\boldsymbol{\mu}$ and covariance $\boldsymbol{\Sigma}$.

The Kalman filter, as every Bayes filter, consists of two steps: first it makes a prediction of the current state distribution, given the previous one:

$$\tilde{\boldsymbol{\mu}}_t = \mathbf{A}\boldsymbol{\mu}_{t-1} + \mathbf{B}\mathbf{u}(t-1) + \mathbf{e} \tag{10}$$

$$\tilde{\boldsymbol{\Sigma}}_t = \mathbf{A}\tilde{\boldsymbol{\Sigma}}_{t-1}\mathbf{A}^T + \mathbf{V}, \tag{11}$$

where $\mathbf{V}$ denotes the process noise and $\mathbf{W}$ the measurement noise.

Second, the Kalman filter updates the prediction $(\tilde{\boldsymbol{\mu}}_t, \tilde{\boldsymbol{\Sigma}}_t)$ given the current measurement $\mathbf{z}_t$ to compute the state distribution $(\boldsymbol{\mu}_t, \boldsymbol{\Sigma}_t)$:

$$\mathbf{K}_t = \tilde{\boldsymbol{\Sigma}}_t\mathbf{C}^T(\mathbf{C}\tilde{\boldsymbol{\Sigma}}_t\mathbf{C}^T + \mathbf{V})^{-1} \qquad \text{(Kalman gain)} \tag{12}$$

$$\boldsymbol{\mu}_t = \tilde{\boldsymbol{\mu}}_t + \mathbf{K}_t(\mathbf{z}_t - \mathbf{C}\tilde{\boldsymbol{\mu}}_t) \tag{13}$$

$$\boldsymbol{\Sigma}_t = (\mathbf{I} - \mathbf{K}_t\mathbf{C})\tilde{\boldsymbol{\Sigma}}_t, \tag{14}$$

where $\mathbf{I}$ denotes the identity matrix.

We can then use the maximum likelihood state hypothesis $\boldsymbol{\mu}_t$ as the estimate of the current state $\mathbf{x}(t)$ when applying LQR.

5 Extended Kalman Filter

The Kalman filter can only be applied if the measurement and process models are linear. In the case the models are nonlinear but differentiable, we can resort to a nonlinear variant of the Kalman filter (Section 4), the *extended Kalman filter (EKF)* [4]. Similar to iLQR, an EKF computes local linearizations of models based on the current state. To deal with a nonlinear process model $f(\mathbf{x}, \mathbf{u})$, we modify the prediction step (Eq. 10) as follows:

$$\tilde{\boldsymbol{\mu}}_t = T(\boldsymbol{\mu}_{t-1}, \mathbf{u}_{t-1}) \tag{15}$$

$$\tilde{\boldsymbol{\Sigma}}_t = \mathbf{J}_T(\boldsymbol{\mu}, \mathbf{u}_{t-1})\tilde{\boldsymbol{\Sigma}}_{t-1}\mathbf{J}_T(\boldsymbol{\mu}, \mathbf{u}_{t-1})^T + \mathbf{V}, \tag{16}$$

where $\mathbf{J}_T = \left(\frac{\partial T_i(\hat{\mathbf{x}}, \hat{\mathbf{u}})}{\partial x_j}\right)_{i,j\in\{1,\ldots,N\}}$ is the Jacobian matrix of the nonlinear dynamics T at state $\hat{\mathbf{x}}$ and action $\hat{\mathbf{u}}$, with N being the state dimensionality. In case f is linear, $\mathbf{J}_T = \mathbf{A}_t$.

To deal with non-linear observation models $c(\mathbf{x})$, we adapt the update step (Eq. 12) as follows:

$$\mathbf{K}_t = \tilde{\boldsymbol{\Sigma}}_t\mathbf{J}_c(\tilde{\boldsymbol{\mu}}_t)^T(\mathbf{J}_c(\tilde{\boldsymbol{\mu}}_t)\tilde{\boldsymbol{\Sigma}}_t\mathbf{J}_c(\tilde{\boldsymbol{\mu}}_t)^T + \mathbf{V})^{-1} \qquad \text{(Kalman gain)} \tag{17}$$

$$\boldsymbol{\mu}_t = \tilde{\boldsymbol{\mu}}_t + \mathbf{K}_t(\mathbf{z}_t - c(\tilde{\boldsymbol{\mu}}_t)) \tag{18}$$

$$\boldsymbol{\Sigma}_t = (\mathbf{I} - \mathbf{K}_t\mathbf{J}_c(\tilde{\boldsymbol{\mu}}_t))\tilde{\boldsymbol{\Sigma}}_t, \tag{19}$$

where $\mathbf{J}_c = \left(\frac{\partial c_i(\hat{\mathbf{x}})}{\partial x_j}\right)_{i,j\in\{1,\ldots,N\}}$ is the Jacobian matrix of c at state $\hat{\mathbf{x}}$.

References

1. Bertsekas DP. Dynamic Programming & Optimal Control. 3rd ed. Belmont, MA, USA: Athena Scientific; 2005.
2. Li W, Todorov E. Iterative linear quadratic regulator design for nonlinear biological movement systems. In: ICINCO (1); 2004. p. 222–229.
3. Todorov E, Li W. A generalized iterative LQG method for locally-optimal feedback control of constrained nonlinear stochastic systems. In: Proceedings of the 2005, American Control Conference. vol. 1. Portland, OR, USA; 2005. p. 300–306.
4. Thrun S, Burgard W, Fox D. Probabilistic Robotics. Cambridge, MA: MIT Press; 2005.
